# Managerial (dis)preferences towards employees working from home: Post-pandemic experimental evidence

**DOI:** 10.1371/journal.pone.0303307

**Published:** 2024-05-15

**Authors:** Agnieszka Kasperska, Anna Matysiak, Ewa Cukrowska-Torzewska

**Affiliations:** Faculty of Economic Sciences, University of Warsaw, Warsaw, Poland; Asia University, TAIWAN

## Abstract

Work from home (WFH) has been a part of the professional landscape for over two decades, yet it was the COVID-19 pandemic that has substantially increased its prevalence. The impact of WFH on careers is rather ambiguous, and a question remains open about how this effect is manifested in the current times considering the recent extensive and widespread use of WFH during the pandemic. To answer these questions, this article investigates whether managerial preferences for promotion, salary increase and training allowance depend on employee engagement in WFH. We take into account the employee’s gender, parental status as well as the frequency of WFH. Furthermore, we examine whether managers’ experience with WFH and its prevalence in the team moderate the effect of WFH on careers. An online survey experiment was run on a sample of over 1,000 managers from the United Kingdom. The experiment was conducted between July and December 2022. The findings indicate that employees who WFH are less likely to be considered for promotion, salary increase and training than on-site workers. The pay and promotion penalties for WFH are particularly true for men (both fathers and non-fathers) and childless women, but not mothers. We also find that employees operating in teams with a higher prevalence of WFH do not experience negative career effects when working from home. Additionally, the more WFH experience the manager has, the lesser the career penalty for engaging in this mode of working. Our study not only provides evidence on WFH and career outcomes in the post-pandemic context but also furthers previous understanding of how WFH impacts careers by showing its effect across different groups of employees, highlighting the importance of familiarisation and social acceptance of flexible working arrangements in their impact on career outcomes.

## Introduction

The incidence of work from home (WFH), broadly defined as conducting work from one’s home rather than the employer’s premises, has been steadily increasing over the last two decades thanks to the development of information communication technologies (ICTs) [1]. The need for social isolation during the COVID-19 pandemic meant that WFH became a central element of professional life for many employees, and its prevalence has sharply increased. In 2019, just before the pandemic, the share of employees who WFH (‘usually’ or ‘sometimes’) in Europe was 11% [2]. This number doubled when the pandemic started and approximately one in four European workers worked from home during that time. In the UK, where this study is situated, WFH was even more widespread and its prevalence exceeded 40% at the peak of the pandemic in 2021 [3]. There is a prevailing argument that WFH will persist and become a standard practice in the professional realm as a considerable number of employees expresses an interest in continuing to WFH despite a decline in health risks associated with COVID-19 infections [4–6]. This is particularly pronounced among parents who perceive WFH as an opportunity to effectively combine paid employment with caregiving responsibilities [7]. Indeed, the data from the Office for National Statistics (ONS) shows that from January 2022 until February 2023, on average, 35% of employees in the UK indicated working from home at some point in the past seven days [8]. Considering the increasing importance of WFH in the workplace, it is imperative to explore the potential ramifications that this mode of work may entail for workers’ careers.

In this study, we explore whether managerial preferences for promotion, salary increase, and training allowance differ depending on the employees’ engagement in WFH in the post-pandemic context of the UK. In particular, we examine whether WFH carries different career effects for women and men, taking into account their parenthood status and the prevalence of WFH in the team. Despite a sizeable volume of research exploring the link between WFH and career development, for example [9–16], the impact of WFH on careers is still rather ambiguous.

On the one hand, WFH has the potential to boost workers’ careers by increasing their productivity levels. This outcome arises due to several factors: reduction of workplace distractions and interruptions which are common in collocated office environments [17–19], the opportunity to allocate more time towards work instead of commuting [9], and the consequential improvements in job satisfaction, job autonomy, and work-life balance [20–25]. Home-based workers were also found to work more intensely [20, 25, 26], which is possibly driven by their desire to reciprocate for the opportunity to work remotely [27] or increased employer expectations [28]. However, WFH can also lead to unfavourable career consequences as it significantly impairs social interactions and communication. Those who WFH therefore experience less knowledge exchange with their co-workers and managers, fewer mentoring and networking opportunities, and are more likely to feel left out [29–32]. They may also be at risk of worse job visibility due to the lack of their physical presence in the workplace [33, 34]. The home environment may also not be free from distractions, as for example, other family members can interrupt the work of home-based workers, lowering their productivity levels [35]. Additionally, employers may perceive workers who WFH as less or more productive than office-based workers due to the beliefs and attributions that they make regarding employees’ motives for engaging in WFH, ultimately impacting the career opportunities of those who WFH [12, 14, 36].

Several factors can moderate the impact of WFH on workers’ careers, and one of them is the frequency of WFH. The experiences of individuals who engage in WFH more frequently are likely to be different than of those who WFH only sporadically. Indeed, a meta-analysis conducted by Gajendran and Harrison [21] revealed that frequent WFH was associated with lower levels of job satisfaction and autonomy, and poorer co-worker relationship quality. Martinez and Gomez [37] showed also that the more employees were engaging in remote work, the fewer opportunities for training and development they were receiving. A more recent study by Golden and Eddleston [11] indicated that the slower salary growth experienced by American remote workers was particularly visible among frequent users of WFH. Higher levels of WFH during the COVID-19 lockdown were also associated with more social isolation and less organisational identification [38]. Social isolation appeared to be particularly important during the pandemic as evidence suggests that it significantly elevated stress levels, and consequently contributed to a decline in productivity for those working from home [39].

Furthermore, the influence of WFH on careers can vary depending on the worker’s gender or parental status. It has been hypothesised that there are varying reasons why men and women engage in this mode of working, with men doing so to increase productivity and women to better combine paid work with caregiving [40–42]. Consequently, employers may consider women, and mothers in particular, to be less promotion-worthy because their engagement in WFH is driven by self-serving motives (e.g. work-family reconciliation) rather than organisation-serving motives (more intense work or longer working hours) [14]. Women who WFH can also be less productive as previous research showed that their work is often interrupted by children and implies a lot of multitasking while teleworking men are better at separating the work and family spheres [43]. On the other hand, women may be rewarded for continuing to work for pay despite increased demand in the personal domain and be able to work longer hours when working from home due to the time saved on commuting [9]. Men who WFH may also face challenges due to high societal expectations of devoting themselves to work, making deviations from such norms particularly difficult for them and leading to adverse career consequences [44–48]. As a result, gender and parenthood likely play a moderating role in the relationship between WFH and career outcomes, although the specific direction of this influence remains uncertain.

Finally, the impact of WFH on managerial decisions regarding promotion, salary and training may also depend on how common this arrangement is in the work environment and the direct supervisor’s familiarity with this mode of working. This is because the prevalence of WFH and its use by the manager signals the degree of social acceptance and naturalisation of this work arrangement. A higher level of WFH in the immediate professional network of the employee can also imply a greater understanding of effective remote workforce management. Previous research suggests that organisational settings, such as high-performance work culture and the financial implications related to the use of flexible work policies, exert a negative influence on the intentions of employees to participate in flexible work [49]. Similarly, men’s use of parental policies at work has been shown to heavily depend on the behaviour of other colleagues, particularly men [50]. Therefore, a higher prevalence of WFH within a group of close workers and its use by the direct supervisor, indicative of higher social acceptance and familiarity with WFH, have the potential to mitigate the adverse career implications typically associated with this work arrangement.

Previous research examining the relationship between WFH and workers’ career outcomes has produced inconsistent findings, with some indicating negative effects [11] and others suggesting positive effects [9, 15, 16] of WFH on workers’ opportunities for promotion or salary increases. However, these studies may be subject to sample selection issues as they rely on survey data. For example, if only the most productive and high-performing workers are granted greater flexibility, the positive influence of WFH on career outcomes may be overestimated [18, 22, 26, 51]. Conversely, the negative effects of WFH may also be overestimated if individuals who request this working arrangement are less career-oriented. A few studies have employed experimental designs to mitigate selection bias and they predominantly found detrimental effects of WFH on workers’ career outcomes [12, 13, 32, 52]. These negative effects were stronger for parents than childless workers, but interestingly, they were attenuated for fathers who pursued WFH for childcare-related reasons [12]. However, it is important to note that these studies were conducted before the COVID-19 pandemic when WFH was much less prevalent and socially accepted.

There are several ways in which this article contributes to the literature. First, to the best of our knowledge, our study is the first post-pandemic study that provides evidence of the effects of WFH on managerial preferences and attitudes towards those engaging in this mode of working. The study, therefore, sheds light on how the impact of WFH on careers is manifested in the current times after the extensive and widespread use of WFH during the pandemic. The results of a survey conducted during the pandemic among home-based workers in the UK showed that 84% of them would like to continue working from home once the pandemic ends [4]. This inclination among employees to continue remote work aligns with the recent data concerning the prevalence of home-based work in the UK during the late/post-pandemic period, which indicates that approximately 35% of employees reported engaging in working from home at least once within the past seven days between January 2022 and February 2023 [8]. It can be stated then that WFH has attained a broader scope of adoption within the UK in comparison to the pre-pandemic period, and the circumstances captured in our survey (in the second half of 2022) closely resemble the post-Covid reality in the country. Besides providing evidence on the impact of WFH on workers’ careers in the post-pandemic context, the study also contributes to the literature by providing causal evidence of the effects of WFH on workers’ careers across different populations (i.e. based on worker’s gender and parenthood status) and organisational settings (i.e. the prevalence of WFH in the team). Although a sizeable body of literature has explored the link between WFH and careers, many of the previous studies rely on survey data, which may suffer from sample selection problems, and the few experimental studies conducted on this topic rarely incorporate such a wide range of moderators (individual and group-level) of the impact of WFH on careers. Our approach thus allows us to not only account for the unobserved factors which may confound the relationship between WFH and careers but also to accurately distinguish how various groups of employees are perceived and judged differently when engaging in WFH. Finally, our findings highlight the importance of organisational settings that either foster or hinder social acceptance and familiarisation of flexible working arrangements and ultimately impact the careers of those who engage in them.

## Materials and methods

We investigate the effects of WFH on workers’ career outcomes in the UK by using data from the survey experiment which was pre-registered on the Open Science Framework. All deviations from the pre-registered plan and the study questionnaire are listed in the S1 Appendix. The study protocol was approved by the Ethics Committee of the University of Warsaw. The experiment was run online in the UK between July and December 2022 by an external research company. The participants were paid for participation in surveys in accordance with the rates indicated by the research company.

### Study design

The study design involved a survey experiment, namely, a paired conjoint with a forced answer. Participants were presented with three sets of worker profiles, with two profiles displayed side by side on each of the three pages (i.e. one page after another without the possibility of going back to the previous page). Each profile consisted of seven attributes (working mode, sex, number of children, age, work experience, skills ranking, and performance rating) that were randomly assigned to the profiles. The levels of the attributes are presented in Table 1. For half of the profile pairs, a randomisation process was employed to show the performance ratings, whereas, for the other pairs, the performance rating was intentionally withheld and marked as ’not provided’. After familiarising themselves with the pair of workers’ profiles, participants were asked five questions, namely which employee they would choose for (1) promotion, (2) salary increase (3) training, and which employee they consider to be (4) more competent and (5) more committed to work. The study instructions and examples of workers’ profiles presented to participants are shared in the S1 Appendix. Once the participants compared three pairs of workers’ profiles, they were asked several questions about themselves and their workplace, including questions on the prevalence of WFH in the company and its use by the direct supervisor.

**Table 1:** The list of attributes and their levels.

Attribute	Level
Sex	Female, Male
Number of children younger than 14 years old in the household	0, 1, 3
Age	38, 40, 41
Whether the employee works from home and the extent of it = working mode	none, 2 days per week, 5 days per week
Full-time work experience in the sector in years	8, 13
The ranking of skills (min 1 and max 5) possessed by the employee	social 2 analytical 5, social 4 analytical 1, social 3 analytical 2
Employee’s performance rank	not provided, satisfactory, exceptional

### Sample

The study participants were recruited from an existing online opt-in panel and comprised of managers (i.e. individuals with supervisory responsibilities) based in the UK. The managers at the time of the survey were employed in occupations, in which the share of jobs that can be done at home is at least 50%, as per a study by Dingel and Neiman [53]. This sample restriction was used to avoid a situation in which a manager does not choose a person who WFH for promotion as working from home is not possible in this occupation. The managers worked in companies that employed at least 10 individuals and supervised at least 5 employees. The data is representative in terms of the size and geographical location of the company, as well as the managers’ gender.

The overall number of participants for which the data has been collected amounted to 1,206. From the initial sample, we chose only individuals who met the selection criteria in terms of the time they allocated to complete the experiment module of the survey. We established a cut-off threshold of 29 seconds, with sub-threshold time limits of less than 15 seconds for the first pair of profiles, less than 9 seconds for the second pair, and less than 5 seconds for the third pair. As a result of implementing this restriction, we excluded 269 respondents, resulting in a final sample size of 937 participants and a total of 5,622 data records (937 individuals * 3 * 2 profiles compared).

Our analysis primarily focuses on the subset of records in which the performance rank (profile attribute) was designated as ’not provided’. We contend that such a focus allows us to capture circumstances observed in the ‘real world’, where employers do not have information about the work performance of remote workers and office-based workers and have to make assumptions about it based on past experience or their own presumptions. This restriction does not alter the number of respondents, which remains at 937, but it does impact the number of records by approximately half (as the performance rating was randomly set to ’not provided’ for half of the pairs of profiles). Consequently, our analysis encompasses 937 respondents evaluating 2,804 fictitious worker profiles (records).

The final sample comprises mostly individuals aged 35 and above, holding managerial positions and possessing higher educational qualifications. Additionally, they have at most two children. Within our sample, 38.5% are women, a proportion consistent with the observed percentage of female managers in 2019 LFS data (which is the latest available) for the United Kingdom [2]. The sample is predominantly composed of IT specialists, accountants, and engineers, constituting 54% of all participants. The managers employed in the IT sector account for 23% of the sample, followed by the accounting and finance departments, which constitute 18.4% of the respondents. The participants assume decision-making responsibilities pertaining to employee promotions (69.7%), training (54.5%), evaluation (90.4%), and employment conditions such as remuneration and contract terms (55%). A significant majority of respondents (78%) indicated that they engage in remote work, at least sporadically. Furthermore, among those who work remotely, the majority (52%) do so in a hybrid manner, alternating between office attendance and working from home several times per week. The demographic characteristics of the respondents included in the analysis are shown in Table 2, while Table 3 presents features of the team and company they work in.

**Table 2:** Characteristics of the respondents.

Variable	Mean	Std. Dev.	Variable	Mean	Std. Dev.
**Age**			**Managerial responsibilities (= 1 if yes)**		
18–34	0.139	0.346	Promotion	0.697	0.460
35–44	0.319	0.466	Training	0.545	0.498
45–54	0.279	0.449	Evaluation	0.904	0.295
55+	0.264	0.441	Employment conditions	0.550	0.498
**Tenure (current position)**			**Occupation**		
Less than 5 years	0.314	0.464	Network Manager	0.035	0.184
5–9 years	0.281	0.450	Software Developer or Computer Programmer	0.066	0.249
10–14 years	0.166	0.373	Systems Administrator	0.027	0.161
15–25 years	0.166	0.373	Other IT professional	0.172	0.377
More than 25 years	0.073	0.260	Accountant	0.118	0.323
**Education**			Financial or business analyst	0.038	0.192
Secondary or less	0.072	0.258	Investment or financial advisor	0.016	0.126
Further (college/6th form/A-levels)	0.170	0.376	Retail or personal banker/loan officer	0.016	0.126
Higher (undergraduate, postgraduate)	0.759	0.428	Other Finance professional	0.078	0.268
**Sex (= 1 if female)**	0.385	0.487	Recruiter	0.011	0.103
**Number of children**			Other HR Professional	0.047	0.212
0 (no children)	0.335	0.472	Sales support / Account Manager	0.049	0.216
1 child	0.386	0.487	Artist, graphic artist, visual design specialist	0.012	0.108
2 children	0.210	0.408	Attorney or Lawyer	0.042	0.200
3 and more children	0.068	0.252	Engineer	0.126	0.332
**WFH (= 1 if yes)**	0.782	0.413	Management Consultant	0.041	0.197
**WFH frequency for those who WFH**			Scientific researcher	0.017	0.130
Daily	0.265	0.441	Writer or journalist	0.012	0.108
Several times a week	0.523	0.500	Marketing and related disciplines	0.049	0.216
Several times a month	0.153	0.360	Other	0.0288	0.1674
Less often than st. a month	0.060	0.238			
Number of observations	937				

**Table 3:** Characteristics of respondents’ team and company.

Variable	Mean	Std. Dev.	Variable	Mean	Std. Dev.
**Team**					
**Department**			**Number of employees (team)**		
Accounting / Finance	0.184	0.387	5–9	0.458	0.498
Administration	0.013	0.113	10–19	0.322	0.468
Business Analytics	0.019	0.137	20–49	0.154	0.361
Customer Relations	0.011	0.103	50–99	0.042	0.200
Engineering	0.100	0.301	>100	0.025	0.155
HR	0.049	0.216	**Share of employees who WFH**		
IT	0.233	0.423	none	0.162	0.369
Legal	0.037	0.190	<20%	0.112	0.316
Management	0.100	0.301	20%-39%	0.086	0.281
Marketing	0.036	0.187	40%-59%	0.064	0.245
Operations	0.055	0.229	60%-79%	0.070	0.256
Promotion / PR	0.010	0.098	>80%	0.505	0.500
Research and development	0.037	0.190	**Manager’s WFH frequency**		
Sales	0.055	0.229	Daily	0.207	0.405
Other	0.060	0.237	Several times a week	0.409	0.492
			Several times a month	0.119	0.325
			Less often	0.047	0.212
			Never	0.218	0.413
**Company**					
**Region**			**Sector**		
North East	0.029	0.167	Manufacturing	0.110	0.313
North West	0.101	0.302	Electricity, Gas, Steam, and Air Conditioning Supply	0.021	0.145
Yorkshire and the Humber	0.073	0.260	Water Supply; Sewerage, Waste Management	0.011	0.103
East Midlands	0.060	0.237	Construction	0.047	0.212
West Midlands	0.080	0.272	Wholesale and Retail Trade; Repair of motor vehicles	0.038	0.192
East of England	0.073	0.260	Transportation and Storage	0.031	0.173
London	0.238	0.426	Accommodation and Food Service Activities	0.013	0.113
South East	0.145	0.352	Information and Communication	0.142	0.349
South West	0.085	0.280	Financial and Insurance Activities	0.209	0.407
Wales	0.035	0.184	Real Estate Activities	0.013	0.113
Scotland	0.081	0.273	Professional, Scientific and Technical Activities	0.100	0.301
**Company size**			Administrative and Support Service Activities	0.012	0.108
10 to 19	0.091	0.287	Public Administration and Defence; Compulsory Social Security	0.033	0.179
20 to 34	0.114	0.318	Education	0.036	0.187
35 to 49	0.085	0.280	Human Health and Social Work Activities	0.031	0.173
50 to 99	0.052	0.223	Arts, Entertainment and Recreation	0.028	0.164
100 to 249	0.084	0.278	Other	0.125	0.331
250 to 499	0.084	0.278			
500 to 999	0.083	0.276			
> 1,000	0.406	0.491			
Number of observations	937				

The participants predominantly work in companies engaged in financial and insurance activities, as well as the information and communications sector. This likely contributes to the significant proportion of employees within the respondents’ teams who engage in remote work. Specifically, in approximately half of our sample (50.5%), over 80% of team members work from home at least occasionally. Conversely, in 16.2% of teams, no team member engages in remote work. Furthermore, around 23.8% of the companies represented in the survey are situated in London. Interestingly, despite the majority of companies being large organisations with over 1,000 employees (40.6%), the managers included in our sample primarily oversee relatively small teams of 5–9 individuals (45.8% on average).

### Key variables

In this study, we explore the effect of WFH on career outcomes. We thus focus on three outcome variables, namely being chosen for (yes or no): (1) promotion, (2) salary increase and (3) training. Our main explanatory variable is the working mode (full-time; 5 days a week), which assumes one of the three categories: working fully on-site (working from the office five days a week), working in a hybrid mode (working two days from home, three days from the office) and fully from home (working from home 5 days a week). Further variables of interest include worker’s sex (coded as women, or men) and parenthood status—number of children in the household (coded as parents, or non-parents). Another moderator variable included in the study is the prevalence of WFH in the team measured by the question ‘How many of the workers under your supervision work from home at least one day a week on a regular basis?’ with three categories: 0–39% (Low), 40–79% (Moderate) and 80+% (High). The final moderator, the manager’s frequency of WFH, is recoded from two measures asking about whether the managers work from home and, if so, how often they do so. The variable assumes three levels: Never, Sporadically (several times a month or less often) and Often (several times a week or daily). Remaining profile attributes, such as workers’ age, work experience and skills are considered to be control variables in the models.

### Data analysis

In our analysis, we employ a logistic regression with a separate model constructed for each of the three outcome variables. The estimated coefficients of the models are used to derive the predicted probabilities (i.e. estimated marginal means) of choosing an employee for promotion, salary raise and training. We use 83% confidence intervals (CIs) as it was demonstrated that nonoverlapping 83% CIs are sufficient to display statistically significant differences (at 0.05 level) between two probabilities in logistic regression [54].

First, we investigate the impact of WFH and its frequency (hybrid vs full-time home-based work) on workers’ opportunities for promotion, salary increase, and training. Subsequently, we examine the potential moderating role of gender and parenthood status by interacting the working mode variable with these factors. Furthermore, we extend our analysis to explore the group-level factors that can influence the effect of WFH on career outcomes. To achieve this, we introduce an additional interaction term involving the prevalence of WFH in the team. This enables us to assess how the career effects of WFH may be contingent upon the level of WFH adoption, and subsequent familiarisation and social acceptance of this mode of working.

## Results

The data analysis results are presented graphically as predicted probabilities, with full regression tables shared in the Tables 1–4 of S1 Appendix.

### Managerial promotion preferences and WFH

**Fig 1:**
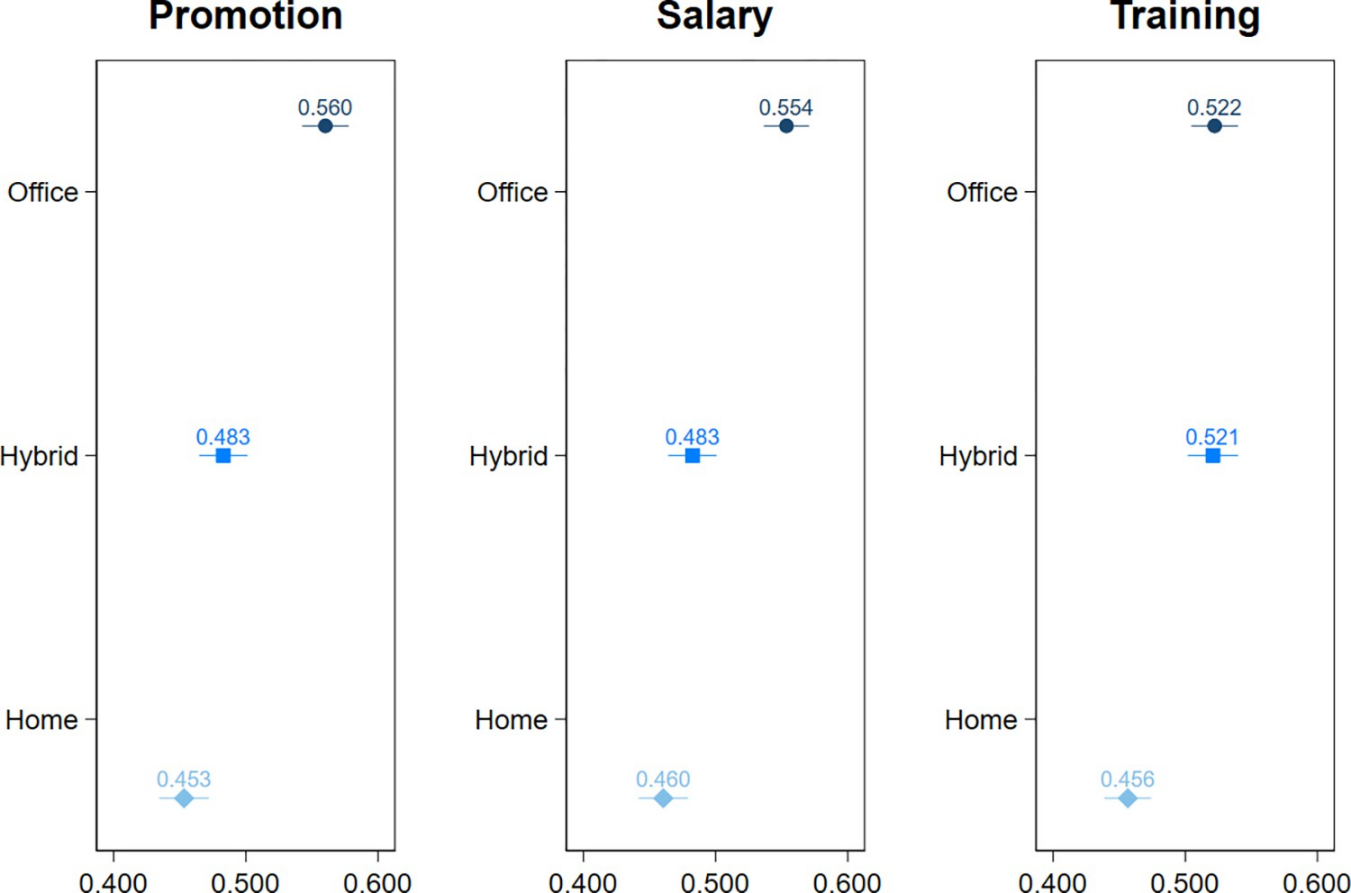
The predicted probabilities for being chosen for promotion, salary increase, and training by working mode: Logit models. Full estimation output is presented in the Table 1 of S1 Appendix. Confidence intervals represent 83%.

First, we explore whether managerial preferences for promotion, salary increase, and training depend on employee engagement in WFH (Fig 1). We find that employees who work in the hybrid and fully home-based mode are less likely to be chosen for promotion and salary increase than those who work from the office. Those, who fully WFH, are also less likely to be chosen for training than office-based workers. Specific results show that those who work in the hybrid mode (2 days at home, 3 days at the office) are 7.7% less likely to be chosen for promotion and 7.1% less likely to be chosen for a salary increase than office-based workers. Those who entirely work from home (5 days at the office) are 10.7% less likely to be chosen for promotion, 9.4% less likely to be chosen for a salary and 6.6% less likely to be chosen for training than those who work on-site. Importantly, there is no statistically significant difference in the chances of being chosen for promotion and salary increase between full-time and hybrid home-based workers though full-time homeworkers are significantly less likely to receive training than hybrid workers. Overall, our findings demonstrate that engaging in WFH carries negative career implications related to diminished chances for promotion, salary increase, and training.

### Moderating effect of the prevalence of WFH in the team

**Fig 2:**
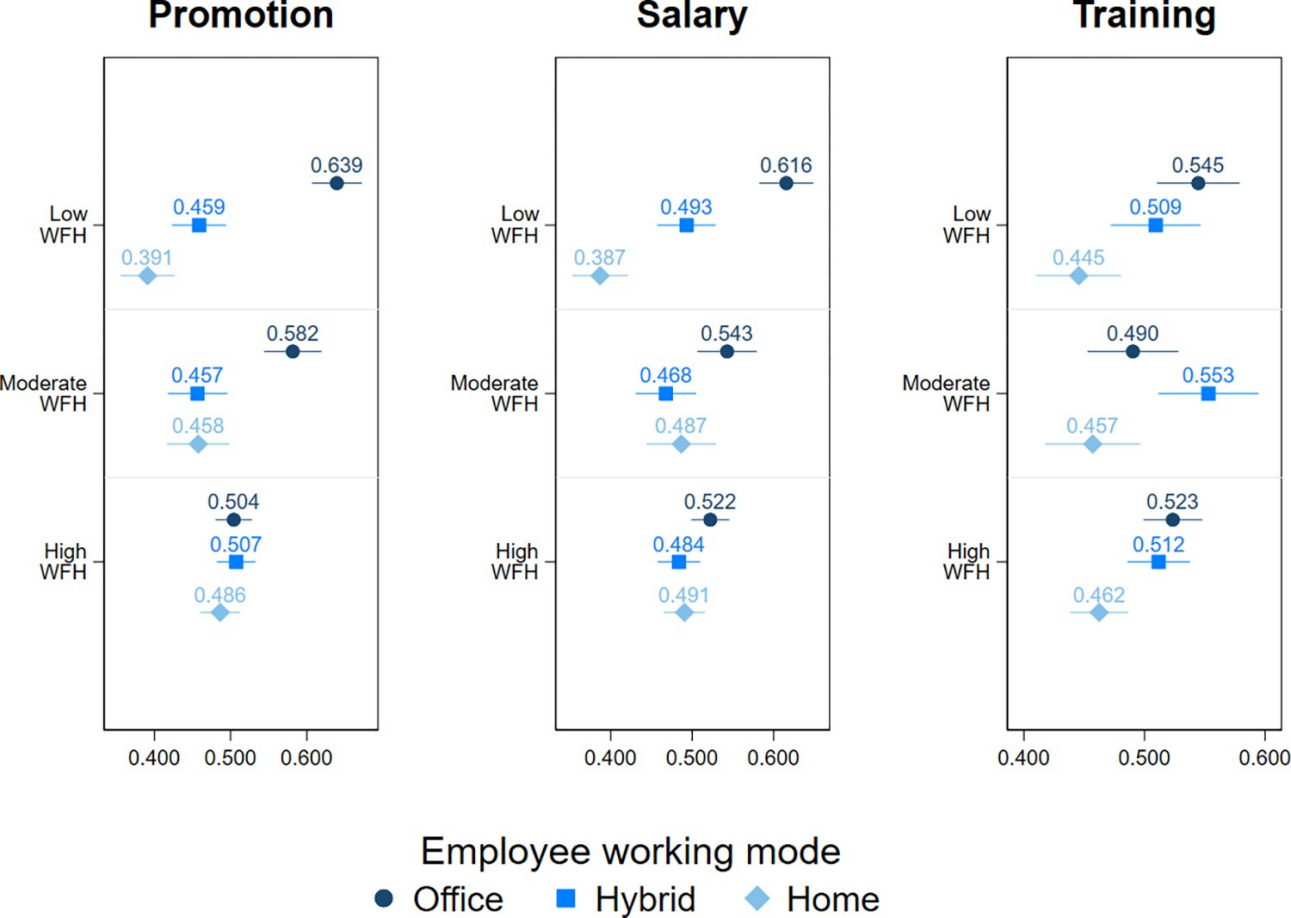
The predicted probabilities for being chosen for promotion, salary increase, and training by working mode and the prevalence of WFH in the team: Logit models. Full estimation output is presented in the Table 2 of S1 Appendix. Confidence intervals represent 83%. WFH prevalence is measured by the question ‘How many of the workers under your supervision work from home at least one day a week on a regular basis?’, with Low WFH referring to 0–39%, Moderate WFH referring to 40–79%, and High WFH referring to more than 80%.

Furthermore, we explore the moderating role of the prevalence of WFH in the team on the impact of WFH on career outcomes (Fig 2). We find that individuals who WFH are less likely to be chosen for promotion and salary increase than those working from the office but only when the prevalence of WFH in their team is lower, namely less than 80% (for promotion) or 40% (for salary increase) of the team members work from home at least one day a week. Clearly, in teams where WFH is common (>80% of workers make use of it), there are no differences in the chances for a promotion or salary increase with respect to the mode of work. Different findings are observed when it comes to training opportunities: here we observe that lower training opportunities are given to full-time home-based workers both when the prevalence of WFH in the team is low (less than 40% of workers use it) and high (more than 80% of workers use it).

### Moderating effect of the manager’s WFH frequency

**Fig 3:**
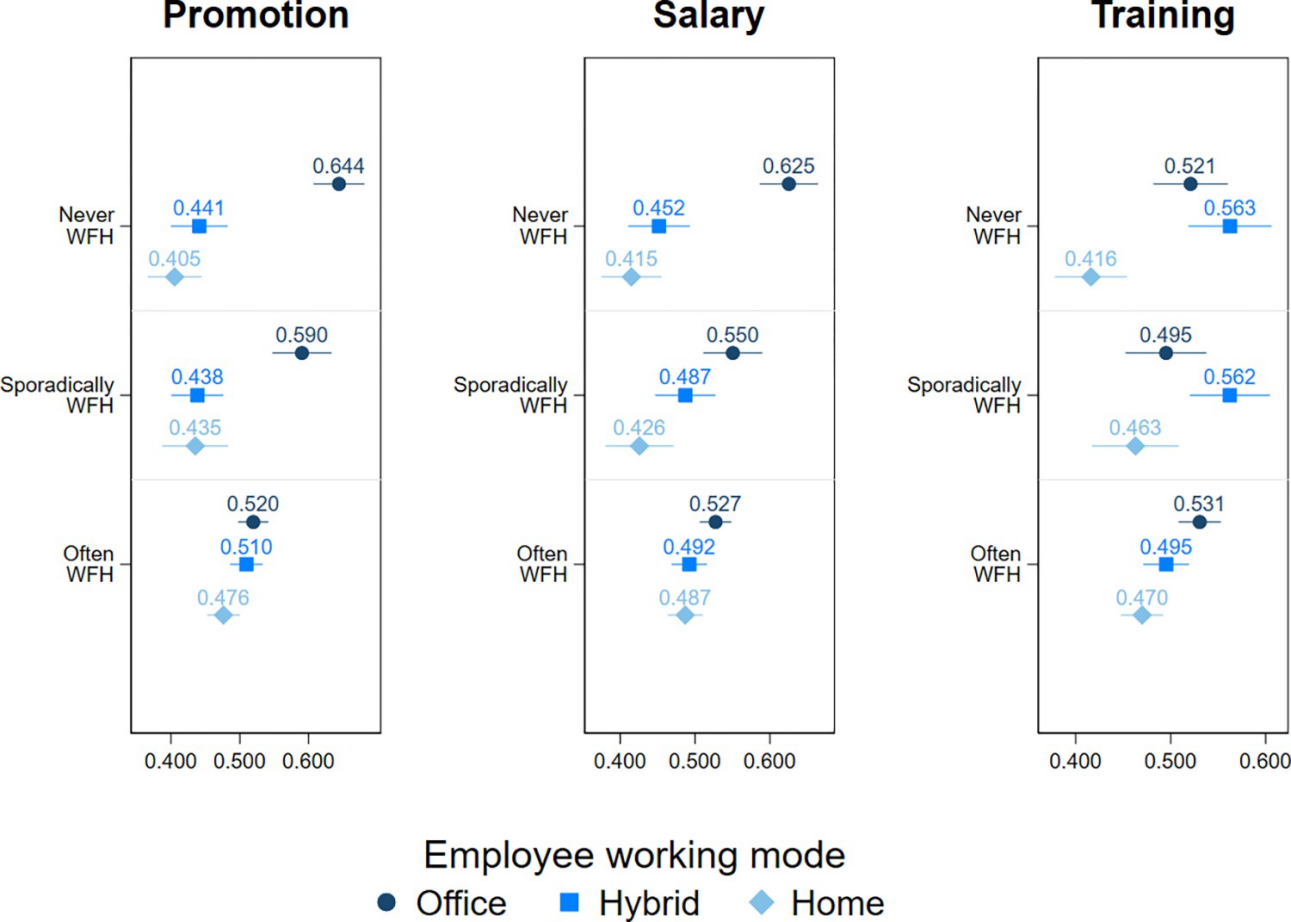
The predicted probabilities for being chosen for promotion, salary increase, and training by working mode and manager’s frequency of WFH: Logit models. Full estimation output is presented in the Table 3 of S1 Appendix. Confidence intervals represent 83%. Manager’s frequency of WFH is measured by the questions ‘Do you currently work from home at least from time to time?’ and ‘How often do you currently work from home?’, with Never, Sporadically referring to several times a month or less often, and Often referring to several times a week or daily.

In the next step, we include the manager’s frequency of WFH as a moderator in the effect of WFH on careers (Fig 3). We find that supervisors who use WFH often (several times a week or daily) are equally inclined to offer promotions and salary raises to employees working from the office as well as those who WFH (both frequencies). However, managers who use WFH sporadically (several times a month or less often) or never are less likely to grant promotions to home-based workers (both frequencies) and salary increases to workers who exclusively WFH. The salary raise penalty is evident among hybrid workers only when the manager never works from home. In general, these findings are largely in line with those on the moderating effects of WFH prevalence in the team, providing robust evidence that more widespread use of WFH in the organisation reduces the promotion and salary penalties for WFH. Regarding training, we find that those who fully WFH are less likely to be granted it when the manager both never works from home and does so often. The results for the sporadic use of WFH by the manager are not statistically significant.

### Moderating effects of gender and parenthood

**Fig 4:**
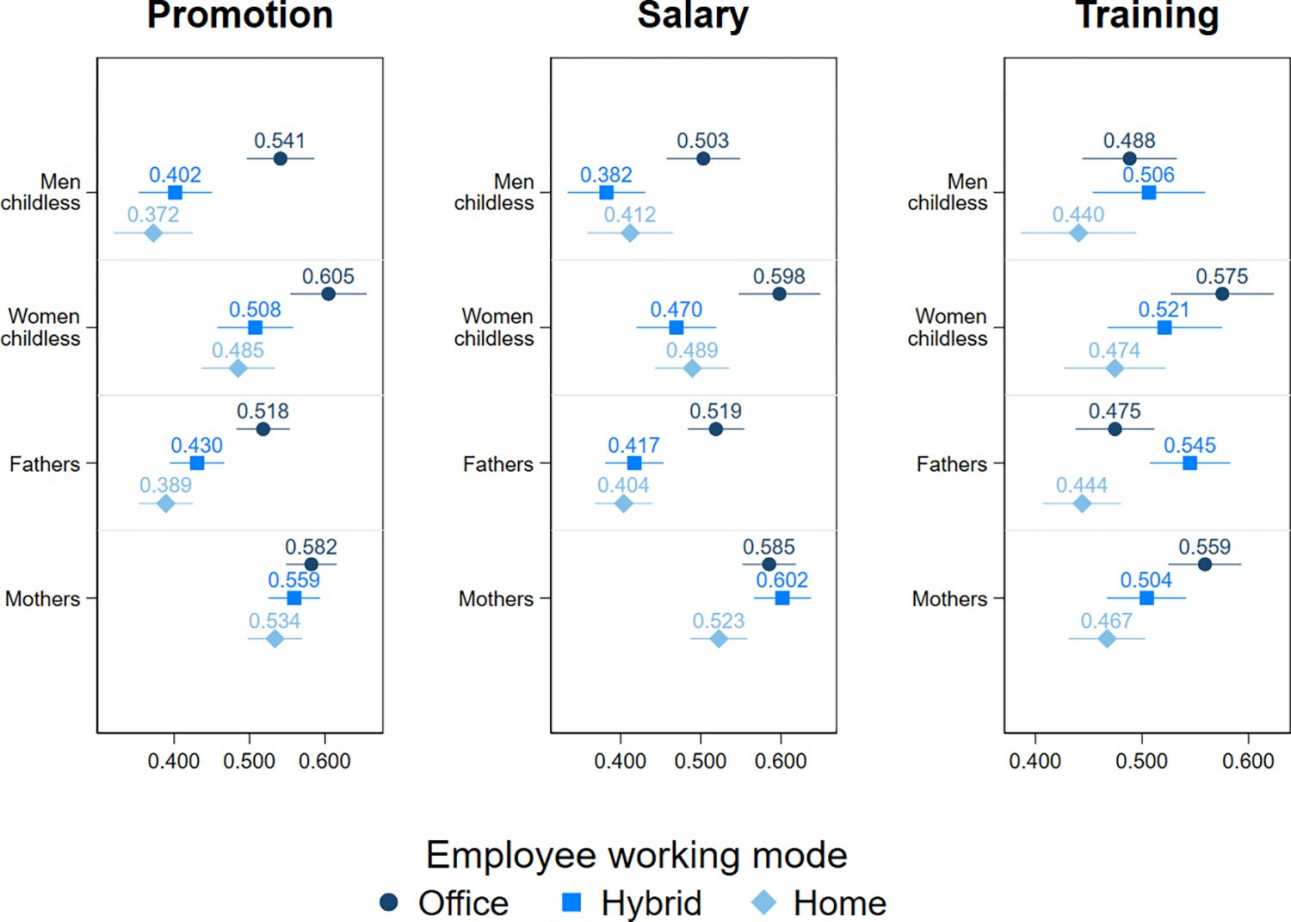
The predicted probabilities for being chosen for promotion, salary increase, and training by working mode, gender and parenthood status: Logit models. Full estimation output is presented in the Table 4 of S1 Appendix. Confidence intervals represent 83%.

Finally, we run interaction models of WFH, gender and parenthood status to explore the moderating role of these variables in the effect of WFH on career progression (Fig 4). Our findings reveal that negative effects of WFH on careers exist for men, both fathers and non-fathers and childless women. Fathers and childless men are less preferred for promotion and salary raises when working from home regardless of their frequency of WFH as both hybrid and fully home-based workers experience similar career consequences (with the exception of childless men who do not get penalised in regards to pay when working fully from home). Whereas, childless women are less likely to be chosen for promotion (but not salary raise) than on-site workers only when they work fully from home. For mothers, we observe no negative consequences of WFH for their promotion or pay, even if they work solely from home. When looking only at the group of fully home-based workers, mothers have higher chances of being preferred for promotion and salary raise than men, both childless men and fathers. Interestingly, in contrast to the first two outcome variables, we find that childless women and mothers who work fully from home are less likely to be chosen for training than on-site workers but we do not find such effects for men. However, it is worth pointing out that the difference in being preferred for training between childless women working from home and those working on-site is marginally statistically significant. In sum, these findings suggest that men and childless women who WFH are less likely to be promoted and receive a pay raise than those working on-site, whereas, mothers working from home are less likely to receive training than on-site working mothers.

In addition, we run the above model in interaction with the prevalence of WFH in the team and we find that negative WFH effects for promotion and pay exist only in teams where the prevalence of WFH is low (i.e. less than 40% of workers WFH at least one day a week). This applies to all workers regardless of their gender or parenthood status (see the Figs 1–3 and Table 5 in S1 Appendix). Consistent findings are obtained when considering the moderator manager’s WFH frequency, with promotion and pay penalties experienced by all workers, yet, only when the manager has little exposure to WFH (never or sporadically categories). The results for these models are also presented in Figs 4–6 and Table 6 of S1 Appendix.

## Discussion

The phenomenon of WFH has witnessed a remarkable surge in popularity, emerging as a prevalent practice in numerous professional contexts [5, 6, 8, 55]. This widespread adoption of WFH can be attributed to its perceived advantages, encompassing various beneficial aspects for both employees and employers. Employees stand to gain from the potential enhancement of work-life balance [10, 20, 21, 39, 56, 57], increased flexibility [58], greater autonomy over their tasks [59], and time saved on commuting [60]. Employers, in turn, can reap advantages such as reduced expenses on physical office space and the potential for a more engaged and productive workforce [24, 26, 51, 60–62]. Research conducted before the COVID-19 pandemic has yielded inconclusive findings regarding the impact of WFH on career outcomes. It is vital to note, however, that previous studies frequently relied on survey data, which may be susceptible to endogeneity issues and sample selection bias, with subsequent overestimation or underestimation of the impact of WFH on careers [9, 14, 51, 61]. Additionally, some of these studies focused on results obtained from a single organisation, rather than including the entire working population [11, 37, 63]. Yet, prior research underscored that employer beliefs about why employees WFH and their levels of productivity while doing so are important for the impact of WFH on careers [14].

In this study, we contribute to the existing literature by providing empirical evidence that establishes a causal link between WFH and career consequences, specifically in terms of promotional prospects, salary raises, and training opportunities. Our findings align with earlier experimental investigations conducted before the pandemic showing the detrimental impact of WFH on careers [12, 13, 52]. Thus, this means that the extensive experience with WFH during the COVID-19 pandemic, and the subsequent normalisation of this mode of working in the public sphere, has not (yet) altered the negative effects of WFH. This is consistent with findings of studies conducted during the COVID-19 pandemic, showing a negative link between WFH and various work-related dimensions, for example, social isolation and interruptions, attachment to the company and knowledge exchange [30–32, 38]. However, contrary to previous research based on survey data [11, 34], we find that the detrimental consequences of WFH on career outcomes are not contingent upon the frequency of remote work, with few exceptions mentioned in the Results sections. Both individuals engaged in hybrid work arrangements (i.e. work 2 days at home and 3 days a week at the office) and those exclusively working from home (i.e. work from home 5 days a week) encounter comparable and diminished prospects for promotion and salary increases. The tendency of managers to exhibit a diminished preference for promoting workers who WFH can be attributed to several factors. One such factor pertains to the challenge faced by managers in accurately evaluating the productivity of employees who WFH, as they rely on assessing the output of their work rather than ‘face time’ which is easier to determine [35, 59]. As a result, managers may exhibit a perceptual bias toward employees who engage in WFH [64]. Moreover, the complexity and ambiguity inherent in coordinating, monitoring, and controlling processes within teleworking teams contribute to decreased operational efficiency, thereby diminishing managers’ preference for promoting remote workers to higher-rank roles [32, 65, 66]. Future research should aim at identifying and investigating the mechanisms behind the reduced propensity among managers to grant home-based or hybrid workers promotions, salary raises and training.

This study also explored whether a manager’s experience with WFH and its prevalence in the team moderate the negative effect of WFH on careers. The findings reveal that managers with limited exposure to WFH are less inclined to endorse promotions and salary raises for employees engaging in this mode of working. Conversely, managers who frequently WFH themselves refrain from penalising home-based workers. A plausible explanation lies in the fact that, when managers personally adopt WFH, they exhibit less bias towards it and they are more aware of both the challenges and advantages associated with remote work. Consequently, this familiarity with WFH can also position them to be able to more effectively manage a workforce engaged in this mode of working. To the best of our knowledge, this is the first experimental study illustrating how managers’ use of WFH moderates their assessments of career opportunities for employees working remotely. As direct supervisors are often seen as the guards to career advancement—they perform performance appraisals and make suggestions for promotions [67]—this finding holds substantial significance for both the literature and practice. We also find that the higher the prevalence of WFH in the team, the less prominent the negative career consequences of WFH. Ultimately, in teams where the share of employees who WFH at least occasionally on a regular basis is 80% or more, there are no differences in chances for promotion, pay and training with respect to the mode of working. This could be because a higher prevalence of WFH in the team may indicate a wider social acceptance and familiarisation with this mode of working. Our results are consistent with previous research in the area [47, 49, 68, 69], indicating the importance of workplace settings in shaping the experience of employees who use flexible working arrangements. However, past research on this topic was often restricted to the level of the whole organisation rather than the co-workers. Therefore, our research further contributes to the literature by demonstrating the influence of the immediate social group at work (i.e. the employee team) on the career outcomes of individuals who WFH.

Furthermore, we provide evidence that managerial (dis)preferences towards employees working from home are gendered and depend on workers’ parental status. Interestingly, we find no negative promotion or pay implications for mothers who WFH, which is true for those who work in both the hybrid mode and solely from home. Employees who experience lower promotion and salary raise prospects when working from home are men (both childless and parents) and childless women. One possible explanation for this finding is that employees engaging in WFH deviate from the stringent norm of an ideal worker who is fully devoted to work, often able to work long hours and puts work above other responsibilities and personal life [70, 71]. Breaking such norms can lead to the stigmatisation of employees and a negative perception of their work, job commitment or productivity [70]. Considering that flexible working arrangements are commonly used by working parents as a means to effectively integrate their professional and personal lives, it can be argued that gender norms and beliefs play a role in shaping the occurrence and targets of flexibility stigma [64]. In the case of this study, when mothers choose to WFH, they deviate from the expectations of the ideal worker, yet align with the prescriptive societal gender norms that women should prioritise family responsibilities over professional pursuits [72]. Whereas, men who engage in WFH deviate from both workplace and societal gender norms, which can lead to unfavourable career outcomes [49]. Indeed, previous studies have consistently demonstrated that men who assume caregiving responsibilities at the expense of work, such as taking parental leave or reducing their working hours, face more severe professional repercussions than women [44–48].

Another important explanation for the finding that childless women and men are more likely to experience negative career consequences for WFH than mothers may be that they are perceived as groups of workers that do not have an important reason to WFH, as compared to mothers who need to combine the increased demand in their personal life with paid work [10, 42, 43]. Consequently, employers may create negative presumptions about their productivity and commitment to work [12, 14, 36]. Within the context of the UK, combining paid work with childrearing is largely a woman’s job as public support for working parents is limited and mothers are seen as primary caregivers [73]. In this country there is also a strong and expanding right to request flexible working and such arrangements are most prevalent among working mothers [1, 74, 75]. This could indicate that, within the UK context, the use of flexible working arrangements may be perceived by managers as more acceptable (and justified) among mothers, as opposed to non-parents and fathers. The strong presence of ideal worker norms in the UK may further disadvantage men and childless women who deviate from them by engaging in flexible work, potentially signalling to their employers a lower commitment to work or productivity [64]. Indeed, the fear of negative career consequences was stated as one of the most important reasons why workers do not take up flexible working arrangements in the UK [64]. Considering that promotion and salary raises are seen as investments in employees [76], men and childless women who decide to WFH may, therefore, be perceived in a negative light as less advancement-worthy employees.

Moreover, it may be reasonable for managers to anticipate that working mothers who engage in WFH will display increased commitment and exert greater effort as a means of reciprocating for the flexibility afforded to them [27, 77]. The use of WFH by employees is often motivated by the desire (or need) to better integrate personal and professional spheres, particularly for individuals with childcare responsibilities [40–42, 74]. For working mothers, the option to WFH may be perceived by managers as an important employee-related benefit allowing them to better reconcile work and family lives. Consequently, managers may hold the belief that mothers who WFH will exhibit an increased commitment to work and productivity to reciprocate for this benefit. Previous research confirms that employees granted the opportunity to work flexibly are often willing to make sacrifices, such as altering the number of hours worked, even at the expense of their personal time or compensation [78]. Additionally, Kelliher and Anderson (2010) find that employees engaged in remote work demonstrate increased effort and heightened commitment. Notably, as evidenced in the mentioned study, trading flexibility for increased effort was not openly discussed or negotiated with the employer, but it was rather entirely assumed by remote workers, which indicates a significant inner desire to reciprocate for this benefit. Prior research also suggests that some managers deliberately exercise their discretion in granting remote work to encourage longer working hours and foster greater commitment [79]. In sum, managers may presume that mothers who engage in WFH feel obliged to reciprocate for the “privilege” to WFH and anticipate that they will fulfil this obligation by delivering additional effort that benefits the employer. As a result, mothers who WFH are not penalised for their participation in this working arrangement.

What is more, in alignment with previous research [37], our findings demonstrate that individuals who WFH receive comparatively less training allowance than on-site workers. However, when considering gender and parenthood, our results substantially diverge from the findings concerning promotion and pay outcome variables presented in this study. We observe that mothers who WFH are less preferred for training opportunities compared to on-site working mothers. Interestingly, these negative effects are not observed for men (results for childless women are marginally statistically significant), which stands in opposition to the findings obtained for promotion and pay raises. We interpret these findings by considering that although training can be seen as a discretionary resource [76, 80], managers from our study may have viewed it primarily as a means of employee ‘improvement’ rather than as a reward. Consequently, those who WFH and are already equally worthy of promotion and pay raise as office-based workers (i.e. mothers) do not need the additional training and development opportunities. In addition, managers may perceive mothers who WFH to be more committed to their work and work harder to reciprocate for being allowed to work flexibly [27, 77]. Therefore, from the managers’ perspective, mothers working from home may be seen as more productive and committed than on-site working mothers, which means that they do not experience negative WFH effects in relation to promotion and pay increases, and are less likely to be granted training as they do not require further improvement to their performance.

In summary, our findings indicate that WFH has detrimental effects on career outcomes in the post-pandemic era. However, it is plausible that these adverse consequences may attenuate over time as WFH becomes even more prevalent and socially acceptable, and employers develop effective mechanisms for managing and evaluating remote workers. Our research results have implications beyond the specific context of this study, as we demonstrate that the adverse effects of WFH on career outcomes are mitigated in settings where this mode of working is more prevalent and when the direct supervisor has more experience with WFH. Given the substantial adoption of WFH in the UK compared to other countries, it is reasonable to anticipate similar career penalties in countries with a lower prevalence of remote work.

## Supporting information

S1 Appendix Appendix–the supplementary information. (PDF)

S1 Data Dataset. (DTA)

## References

[1] Eurofound. The rise in telework: Impact on working conditions and regulations. Luxembourg: Publications Office of the European Union; 2022.

[2] Eurostat. The European Union Labour Force Survey (EU-LFS); 2019 [cited 2023 June]. Eurostat [Internet]. Available from: https://ec.europa.eu/eurostat/web/microdata/european-union-labour-force-survey

[3] Eurostat. The European Union Labour Force Survey (EU-LFS); 2023 [cited 2023 June]. Eurostat [Internet]. Available from: https://ec.europa.eu/eurostat/data/database?node_code=lfsa_ehomp

[4] Office for National Statistics (ONS). Is hybrid working here to stay?; 2022 [cited 2022 May 23]. Available from: https://www.ons.gov.uk/employmentandlabourmarket/peopleinwork/employmentandemployeetypes/articles/ishybridworkingheretostay/2022-05-23

[5] Barrero JM, Bloom N, Davis SJ. Why working from home will stick. Natl Bureau Econ Res. 2021. Report No.: w28731.

[6] Ozimek A. The future of remote work. Working Paper Available at SSRN. 2020 Jun 24. Report No.: 3638597.

[7] Thompson RJ, Payne SC, Alexander AL, Gaskins VA, Henning JB. Correction to: A Taxonomy of Employee Motives for Telework. Occup Health Sci. 2022;6(2):179–182. 10.1007/s41542-021-00094-5 PMC8415699 34514089

[8] Office for National Statistics (ONS). Characteristics of homeworkers, Great Britain: September 2022 to January 2023. 2023 [cited 2023 June]. Available from: https://www.ons.gov.uk/employmentandlabourmarket/peopleinwork/employmentandemployeetypes/articles/characteristicsofhomeworkersgreatbritain/september2022tojanuary2023

[9] Arntz M, Yahmed SB, Berlingieri F. Working from Home, Hours Worked and Wages: Heterogeneity by gender and parenthood. Labour Economics. 2022;76:102169.

[10] Chung H, Van der Lippe T. Flexible working, work-life balance, and gender equality: Introduction. Soc Indic Res. 2020;151(2):365–381. doi: 10.1007/s11205-018-2025-x 33029036 PMC7505827

[11] Golden TD, Eddleston KA. Is there a price telecommuters pay? Examining the relationship between telecommuting and objective career success. J Vocat Behav. 2020;116:103348.

[12] Munsch CL. Flexible work, flexible penalties: The effect of gender, childcare, and type of request on the flexibility bias. Soc Forces. 2016;94(4):1567–1591.

[13] Bloom N, Liang J, Roberts J, Ying ZJ. Does working from home work? Evidence from a Chinese experiment. Q J Econ. 2015;130(1):165–218.

[14] Leslie LM, Manchester CF, Park TY, Mehng SA. Flexible work practices: a source of career premiums or penalties? Acad Manage J. 2012;55(6):1407–1428.

[15] Heywood JS, Siebert WS, Wei X. The implicit wage costs of family friendly work practices. Oxf Econ Pap. 2007;59(2):275–300.

[16] Weeden KA. Is there a flexiglass ceiling? Flexible work arrangements and wages in the United States. Soc Sci Res. 2005;34(2):454–482.

[17] Aczel B, Kovacs M, Van Der Lippe T, Szaszi B. Researchers working from home: Benefits and challenges. PloS one. 2021;16(3):e0249127. doi: 10.1371/journal.pone.0249127 33765047 PMC7993618

[18] Chen Y, Weziak-Bialowolska D, Lee MT, Bialowolski P, Cowden RG, McNeely E, et al. Working from home and subsequent work outcomes: Pre-pandemic evidence. PLoS One. 2023;18(4):e0283788. doi: 10.1371/journal.pone.0283788 37014892 PMC10072379

[19] Korbel JO, Stegle O. Effects of the COVID-19 pandemic on life scientists. Genome biology. 2020;21(1):1–5. doi: 10.1186/s13059-020-02031-1 32393316 PMC7212246

[20] Felstead A, Henseke G. Assessing the growth of remote working and its consequences for effort, well-being and work-life balance. New Technol Work Employ. 2017;32(3):195–212.

[21] Gajendran RS, Harrison DA. The good, the bad, and the unknown about telecommuting: Meta-analysis of psychological mediators and individual consequences. J Appl Psychol. 2007;92(6):1524–1541. doi: 10.1037/0021-9010.92.6.1524 18020794

[22] Angelici M, Profeta P. Smart working: work flexibility without constraints. Management Science. 2024;70(3):1680–1705.

[23] Chu AM, Chan TW, So MK. Learning from work-from-home issues during the COVID-19 pandemic: Balance speaks louder than words. PloS One. 2022;17(1):e0261969. doi: 10.1371/journal.pone.0261969 35025893 PMC8758108

[24] Gajendran RS, Ponnapalli AR, Wang C, Javalagi AA. A dual pathway model of remote work intensity: A meta-analysis of its simultaneous positive and negative effects. Personnel Psychology. 2024.

[25] Boca D, Daniela NO, Profeta P, Rossi M. Women’s and Men’s Work, Housework and Childcare, before and during COVID-19. Review of Economics of the Household. 2020;18(4):1001–1017. doi: 10.1007/s11150-020-09502-1 32922242 PMC7474798

[26] Deole SS, Deter M, Huang Y. Home sweet home: Working from home and employee performance during the COVID-19 pandemic in the UK. Labour Economics. 2023;80:102295. doi: 10.1016/j.labeco.2022.102295 36440260 PMC9678226

[27] Kelliher C, Anderson D. Doing more with less? Flexible working practices and the intensification of work. Hum Relat. 2010;63(1):83–106.

[28] Wang B, Liu Y, Qian J, Parker SK. Achieving effective remote working during the COVID-19 pandemic: A work design perspective. Applied psychology. 2021;70(1):16–59. doi: 10.1111/apps.12290 33230359 PMC7675760

[29] Gibbs M, Mengel F, Siemroth C. Work from home and productivity: Evidence from personnel and analytics data on information technology professionals. J Political Economy Microeconomics. 2023;1(1):7–41.

[30] Nesher Shoshan H, Wehrt W. Understanding “Zoom fatigue”: A mixed-method approach. Applied Psychology. 2022;71(3):827–852.

[31] DeFilippis E, Impink SM, Singell M, Polzer JT, Sadun R. Collaborating during coronavirus: The impact of COVID-19 on the nature of work (No. w27612). National Bureau of Economic Research. 2020.

[32] Shen L. Does working from home work? A natural experiment from lockdowns. European Economic Review. 2023;151:104323.

[33] Srivastava M. Work Place Flexibility: Implications for Developmental Opportunities and Work-Family Conflicts. Psychol Stud. 2011;56(3):311–317.

[34] Maruyama T, Tietze S. From anxiety to assurance: concerns and outcomes of telework. Pers Rev. 2012;41(4):450–469.

[35] Demerouti E, Derks D, Ten Brummlhuis LL, Bakker AB. New ways of working, impact on working conditions, work-family balance, and well-being. In: Korunka C, Hoonakker P, eds. The impact of ICT on quality of working life. Dordrecht: Springer; 2014. p. 123–141.

[36] Bourdeau S, Ollier-Malaterre A, Houlfort N. Not All Work-Life Policies Are Created Equal: Career Consequences of Using Enabling Versus Enclosing Work-Life Policies. Acad Manag Rev. 2019;44(1):172–193.

[37] Martinez P, Gómez CB. Trading telecommuting flexibility for fewer training opportunities? Manage Res J Iberoam Acad Manage. 2013;11(3):235–259.

[38] Kossen C, van der Berg AM. When the exception becomes the norm: A quantitative analysis of the dark side of work from home. German Journal of Human Resource Management. 2022;36(3):213–237.

[39] Shirmohammadi M, Au WC, Beigi M. Remote work and work-life balance: Lessons learned from the COVID-19 pandemic and suggestions for HRD practitioners. Human Resource Development International. 2022;25(2):163–181.

[40] Hilbrecht M, Shaw SM, Johnson LC, Andrey J. ’I’m home for the kids’: contradictory implications for work–life balance of teleworking mothers. Gender Work Organ. 2008;15(5):454–476.

[41] Bailey DE, Kurland NB. A review of telework research: Findings, new directions, and lessons for the study of modern work. J Organ Behav. 2002;23(4):383–400.

[42] Sullivan C, Lewis S. Home‐based telework, gender, and the synchronization of work and family: perspectives of teleworkers and their co‐residents. Gender Work Organ. 2001;8(2):123–145.

[43] Powell A, Craig L. Gender differences in working at home and time use patterns: Evidence from Australia. Work Employ Soc. 2015;29(4):571–589.

[44] Evertsson M. Parental leave and careers: Women’s and men’s wages after parental leave in Sweden. Adv Life Course Res. 2016;29:26–40.

[45] Vandello JA, Hettinger VE, Bosson JK, Siddiqi J. When equal isn’t really equal: The masculine dilemma of seeking work flexibility. J Soc Issues. 2013;69(2):303–321.

[46] Rudman LA, Mescher K. Penalizing men who request a family leave: Is flexibility stigma a femininity stigma? J Soc Issues. 2013;69(2):322–340.

[47] Coltrane S, Miller EC, DeHaan T, Stewart L. Fathers and the flexibility stigma. J Soc Issues. 2013;69(2):279–302.

[48] Moss-Racusin CA, Phelan JE, Rudman LA. When men break the gender rules: status incongruity and backlash against modest men. Psychol Men Masc. 2010;11(2):140.

[49] Thébaud S, Pedulla DS. When Do Work-Family Policies Work? Unpacking the Effects of Stigma and Financial Costs for Men and Women. Work Occup. 2022;49(2):229–263.

[50] Dahl GB, Løken KV, Mogstad M. Peer effects in program participation. Am Econ Rev. 2014;104(7):2049–2074.

[51] Glass JL, Noonan MC. Telecommuting and Earnings Trajectories Among American Women and Men 1989–2008. Soc Forces. 2016;95(1):217–250. doi: 10.1093/sf/sow034 27833214 PMC5100676

[52] Fernandez-Lozano I, González MJ, Jurado-Guerrero T, Martínez-Pastor JI. The hidden cost of flexibility: A factorial survey experiment on job promotion. Eur Sociol Rev. 2020;36(2):265–283.

[53] Dingel JI, Neiman B. How many jobs can be done at home? J Public Econ. 2020;189:104235. doi: 10.1016/j.jpubeco.2020.104235 32834177 PMC7346841

[54] Austin PC, Hux JE. A brief note on overlapping confidence intervals. J Vasc Surg. 2002;36(1):194–195. doi: 10.1067/mva.2002.125015 12096281

[55] Kaiser S, Suess S, Cohen R, Mikkelsen EN, Pedersen AR. Working from home: Findings and prospects for further research. German Journal of Human Resource Management. 2022;36(3):205–212.

[56] Laß I, Wooden M. Working from Home and Work–Family Conflict. Work Employ Soc. 2023;37(1):176–195. 10.1177/09500170221080870 PMC9929191 36820233

[57] Kurowska A, Kasperska A, Kaufman G. Work from home and perceived changes to work-life balance among mothers and fathers during the COVID-19 pandemic (Working paper No. 2023–29).

[58] White M, Hill S, McGovern P, Mills C, Smeaton D. ‘High‐performance’ management practices, working hours and work–life balance. British Journal of Industrial Relations. 2003;41(2):175–195.

[59] Kossek EE, Thompson RJ. Workplace flexibility: Integrating employer and employee perspectives to close the research–practice implementation gap. In: Pitt-Catsouphes M, Kossek EE, Sweet S, editors. The Oxford Handbook of Work and Family. Oxford: Oxford University Press; 2016. p. 215–239.

[60] Vega RP, Anderson AJ, Kaplan SA. A within-person examination of the effects of telework. J Bus Psychol. 2015;30:313–323.

[61] Lott Y, Chung H. Gender discrepancies in the outcomes of schedule control on overtime hours and income in Germany. Eur Sociol Rev. 2016;32(6):752–765.

[62] De Menezes LM, Kelliher C. Flexible working and performance: A systematic review of the evidence for a business case. Int J Manag Rev. 2011;13(4):452–474.

[63] Golden T. Co-workers who telework and the impact on those in the office: Understanding the implications of virtual work for co-worker satisfaction and turnover intentions. Hum Relat. 2007;60(11):1641–1667.

[64] Chung H. Gender, flexibility stigma and the perceived negative consequences of flexible working in the UK. Soc Indic Res. 2020;151(2):521–545. 10.1007/s11205-018-2025-x PMC7505827 33029036

[65] Baruch Y. Teleworking: benefits and pitfalls as perceived by professionals and managers. New technology, work and employment. 2000;15(1):34–49.

[66] Van der Lippe T, Lippényi Z. Co‐workers working from home and individual and team performance. New Technol Work Employ. 2020;35(1):60–79. doi: 10.1111/ntwe.12153 32214593 PMC7079547

[67] Greenhaus JH, Callanan GA, Godshalk VM. Career management for life. Routledge; 2018.

[68] Albiston C, O’Connor LT. Just leave. Harv Womens LJ. 2016;39(1).

[69] Lott Y, Abendroth AK. The non-use of telework in an ideal worker culture: Why women perceive more cultural barriers. Community Work Fam. 2020;23(5):593–611.

[70] Williams JC, Blair‐Loy M, Berdahl JL. Cultural schemas, social class, and the flexibility stigma. J Soc Issues. 2013;69(2):209–234.

[71] Cech EA, Blair-Loy M. Consequences of Flexibility Stigma Among Academic Scientists and Engineers. Work Occup. 2014;41(1):86–110.

[72] Blair-Loy M. Competing devotions: Career and family among women executives. Cambridge, US: Harvard University Press; 2003.

[73] Matysiak A, Węziak-Białowolska D. Country-specific conditions for work and family reconciliation: An attempt at quantification. Eur J Popul. 2016;32:475–510. doi: 10.1007/s10680-015-9366-9 27795600 PMC5056952

[74] Chung H, Van der Horst M. Women’s employment patterns after childbirth and the perceived access to and use of flexitime and teleworking. Hum Relat. 2018;71(1):47–72. doi: 10.1177/0018726717713828 29276304 PMC5714156

[75] Wanrooy B, Bewley H, Bryson A. Employment relations in the shadow of recession: Findings from the 2011 workplace employment relations study. Bloomsbury Publishing; 2013.

[76] Gavino MC, Wayne SJ, Erdogan B. Discretionary and transactional human resource practices and employee outcomes: the role of perceived organizational support. Hum Resour Manag. 2012;51(5):665–686.

[77] Belmi P, Pfeffer J. How "organization" can weaken the norm of reciprocity: The effects of attributions for favors and a calculative mindset. Acad Manag Discov. 2015;1(1):36–57.

[78] Golden L. Flexible work schedules: What are we trading off to get them. Monthly Lab Rev. 2001;124:50.

[79] Bathini DR, Kandathil GM. An orchestrated negotiated exchange: Trading home-based telework for intensified work. J Bus Ethics. 2019;154:411–423.

[80] Shore LM, Shore TH. Perceived organizational support and organizational justice. In: Cropanzano RS, Kacmar KM, editors. Organizational Politics, Justice, and Support: Managing the Social Climate of the Workplace. Westport, CT: Quorum; 1995. p. 149–164.

