# Supplementary material for: Managerial (dis)preferences towards employees working from home: Post-pandemic experimental evidence
Source: PLoS One. 2024 May 15;19(5):e0303307. doi: 10.1371/journal.pone.0303307 (PMC11095720; doi:10.1371/journal.pone.0303307)
Supplement: S1 Appendix — (PDF) [file pone.0303307.s001.pdf]

A note shown to the participants before the study:

PAGE 1:

This survey is on the topic of employee evaluation, and the results will be used to inform our client. It is being conducted by the University of Warsaw, Poland.

Your answers will be confidential and entirely anonymous. There are no right or wrong answers – all your answers are valuable and important to us, as they will allow us to learn about your preferences and opinions.

Your Account will be credited with 50 points for completing the survey.

We have tested the survey and found that, on average it takes around 15 minutes to complete. This time may vary depending on factors such as your Internet connection speed and the answers you give.

Please click the forward button below to continue.

PAGE 2:

Imagine it's the time of an internal review process for the employees in your team. This is the period when promotions, training, salary raises, etc. are decided upon. We will now present you three pairs of workers' profiles, prepared by your HR department based on the in-company (between-workers) evaluation of skills and performance. For each pair of profiles, you will be asked to choose a worker to whom you would give a promotion, training, salary increase, etc. Due to budgeting limits, you can grant some of the benefits to only one of the workers in each pair. Please consider your choices carefully.

After evaluating workers' profiles, we will also ask you some questions about yourself and your company. Once you go to the next page, you will not be able to go back.

An example of the pair of worker's profiles:

Please, familiarise yourself with the two profiles and answer the questions below.

	**Worker A**	**Worker B**
**Performance rank** (below satisfactory, satisfactory, exceptional)	not provided	not provided
**Work experience in the sector** (in full-time equivalent)	13 years	8 years
**Family situation** (number of children of age 14 and below)	0 children	3 children
**Working mode** (full-time, 5 days a week)	3 days at office; 2 days at home	5 days at office
**Sex**	men	women
**Skills rank** (1 very weak, 5 very strong)	social 2, analytical 3	social 3, analytical 2
**Age**	40 years old	38 years old

Which employee would you give a promotion to?

Worker A	Worker B

Which employee would you give a salary increase to?

Worker A	Worker B

Which employee would you give training to?

Worker A	Worker B

Which employee do you consider to be more committed?

Worker A	Worker B

Which employee do you consider to be more competent?

Worker A	Worker B

Deviations from the pre-registered plan:

Our study procedure and analysis plan were preregistered on the Open Science Framework https://osf.io/n2byz?view_only=7acb8a6da1c141fc8ca4f4391feea700. The following study pertains to the 'Component 1' section of the pre-registration.

In the pre-registration, we stated the following hypothesis:

H1: The HBW effect is stronger for women than men (Gender effect)

H2: The HBW effect is stronger among parents than non-parents (Parenthood effect)

H3: The parenthood effect is stronger for male than female employees (Fatherhood vs motherhood effect)

However, rather than analysing these effects in separate models (i.e. with the use of sub-groups) as previously indicated, we decided to run a triple interaction model in order to better account for the differences between these groups. In addition, we run all models only on the subsample of records where the performance rank was not revealed to the respondent (rather than in comparison with subsample with known performance rank). We decided to focus on the subsample with unknown performance in the first step to display the effects of WFH which closely resemble the 'real life' situation where managers are not fully aware of the productivity levels of their employers and may have certain pre-assumptions about those who WFH. We will compare the performance effect (known vs. unknown performance) in a second step as a sperate study in which we explore the role of the mechanisms behind the WFH effects observed in the 'real' world. Finally, 'Component 1' section in the pre-registration did not include information on the use of 'WFH prevalence' as a moderator in this study. This does not necessarily constitute a deviation from the original research plan as we stated that we may want to conduct exploratory analysis. However, we believe it is important to point out that the idea to use WFH prevalence as a moderator was born after the pre-registration.

Study questionnaire:

Participants first answered screening questions (sample restrictions are explained in the Data & Methods section). Then they were presented with information on the experiment and instructions (see p. 1 of the Appendix). Subsequently, they were tasked with selecting one employee from each pair for promotion, salary raise, training, and which employee they consider to be more competent and more committed to work. They then answered various questions about themselves and the company that they work within, which are presented in this section below.

Please, answer the following questions about the company that you work in.

Q200. Approximately how many people are employed by your company/employer in the country you live in?

<1> 1 (just me)
<2> 2
<3> 3 to 5
<4> 6 to 9
<5> 10 to 19
<6> 20 to 34
<7> 35 to 49
<8> 50 to 99
<9> 100 to 249
<10> 250 to 499
<11> 500 to 999
<12> 1,000 or more

How many of the people employed in your company (in the country you live in) are women?

<1> less than 20%
<2> 20-39%
<3> 40-59%
<4> 60-79%
<5> 80% or more

Please, indicate which measures are provided in your company (Yes / No / Don't know):

<1> Support with childcare (e.g. company childcare facility, cooperation with childcare facilities, childcare during school holidays) or financial contributions towards childcare
<2> Additional employer-funded childcare-related leave (e.g. maternity/paternity/parental, in case of child's sickness) or additional payment during a statutory leave
<3> Flexible start and finish times
<4> Working-time accounts
<5> Option to work from home
<6> Part-time work
<7> No meetings / events organized after certain time.

Highly successful workers in your company are those who…

<1> Work long hours
<2> Are available to work overtime hours whenever needed
<3> Frequently bring work home to finish uncompleted tasks
<4> Are available beyond working hours (for example, quickly replying to email, phone calls, text messages outside of normal business hours)
<5> Put work above personal life

<6> Often do not take vacations
<7> Do not take time off for family reasons if work needs to be done
<8> Do not call in sick
<9> Often work from home during normal business hours
<10> Often work in the office beyond standard work hours
<11> Often choose to change work hours so that they are different from a standard 9 to 5 schedule

#Scale
<1> Definitely not
<2> Rather not
<3> Rather yes
<4> Definitely yes
<9> Does not apply to my company

Which sector of the economy does your company operate in?

<1> Agriculture, Foresty and Fishing
<2> Mining and Quarrying
<3> Manufacturing
<4> Electricity, Gas, Steam and Air Conditioning Supply
<5> Water Supply; Sewerage, Waste Management and Remediation Activities
<6> Construction
<7> Wholesale and Retail Trade; Repair Of Motor Vehicles and Motorcycles
<8> Transportation and Storage
<9> Accommodation and Food Service Activities
<10> Information and Communication
<11> Financial and Insurance Activities
<12> Real Estate Activities
<13> Professional, Scientific and Technical Activities
<14> Administrative and Support Service Activities
<15> Public Administration and Defence; Compulsory Social Security
<16> Education
<17> Human Health and Social Work Activities
<18> Arts, Entertainment and Recreation
<19> Other Service Activities (What?)
<20> Activities Of Households As Employers; Undifferentiated Goods- and Services-Producing Activities Of Households For Own Use
<21> Activities Of Extraterritorial Organisations and Bodies

Please, answer the following questions about yourself.

What department do you work in?
<1> IT
<2> HR
<3> Legal
<4> Business Analytics
<5> Customer Relations
<6> Promotion / PR
<7> Marketing
<8> Sales

<9> Accounting / Finance
<10> Purchase
<11> Operations
<12> Logistics
<13> Administration
<14> Research and Development (R&D)
<15> Management
<16> Engineering
<14> Other

How many years of work experience do you have (overall)?
<1> less than 10 years
<2> 10-14 years
<3> 15-19 years
<4 > 20-25 years
<5 > 25-29
<6> 30-34
<7> 35 or more

How many years of work experience do you have (current position)?
<1> less than 5 years
<2> 5-9 years
<3> 10-14 years
<4> 15-19 years
<5> 20-25 years
<6> more than 25 years

How many people work under your supervision (approximately)?
<1> 5-9
< 2> 10-19
< 3> 20-49
< 4> 50-99
< 5> more than 100

Q6A. How many of them are female?
<1> less than 35%
<2> 35-65%
<3> more than 65%

How many of them are part-time workers?
<1> less than 35%
<2> 35-65%
<3> more than 65%
<4> no part-time workers

How many of the part-time workers are female?
<1> less than 35%
<2> 35-65%
<3> more than 65%

How many of the workers under your supervision work from home at least one day a week?
<1> none
<2> less than 20%
<3> 20%-39%
<4> 40%-59%
<5> 60%-79%
<6> more than 80%

How many of the workers under your supervision work from home at least one day a week are female?
<1> less than 35%
<2> 35-65%
<3> more than 65%

How many of the workers under your supervision worked from home at least one day a week before the outbreak of the Covid-19 pandemic?
<1> none
<2> less than 20%
<3> 20%-39%
<4> 40%-59%
<5> 60%-79%
<6> more than 80%

Taking into consideration the tasks your employees perform at work, what is the maximum proportion of workers who would be able to do their work from home?
<1> none
<2> less than 20%
<3> 20%-39%
<4> 40%-59%
<5> 60%-79%
<6> more than 80%

Do you think the proportion of workers who currently work from home in your company will eventually:

<1> Decline to the levels observed from before the COVID-19 pandemic
<2> Decline but will be higher than before the COVID-19 pandemic
<3> Will remain as it is now
<4> Will increase

Do you currently work from home at least from time to time?
<1> Yes
<2> No

How often do you currently work from home?
<1> Daily
<2> Several times a week
<3> Several times a month
<4> Less often

What is the major reason for which you currently work from home?
<1> Work-life balance / Family obligations

<2> To finish/do extra work
<3> Social distancing (due to Covid)
<7> To save on commuting time
<8> To be more productive
<9> There is no office I could work at
<4> Other (What?)

In your establishment, are you responsible for…. (Yes / No)?
<1> promoting workers to higher positions
<2> granting training allowances
<3> employee evaluation
<4> changes to employment conditions (e.g. pay increases, contract type)

Please, indicate how much you agree with the following statements.
<1> Men should do as much housework and childcare as women.
<2> A man's primary job is to earn money for the family, not to look after the children.
<3> A woman's primary job is to look after the home and family.
<4> All in all family life suffers when the mother has a full-time job.

#Scale
<1> 1 Strongly disagree
<2> 2
<3> 3
<4> 4
<5> 5 Strongly agree

What is the highest level of your obtained education?
<1> primary
<2> secondary
<3> further (college/6th form/A-levels)
<4> higher (undergraduate, postgraduate)

How important, according to you, are social surveys for understanding social phenomena?
<1> very important
<2> rather important
<3> neither important nor unimportant
<4> rather not important
<5> not important at all

Q15. How important are the following skills, in your opinion, for doing your job?

<1> Analytical
<2> Social

#Scale
<1> Not important
<2> Weakly important
<3> Moderately important
<4> Important
<5> Very important

Thank you for taking part in our survey.
Respondents' demographics (data from panel):

- sex
- age
- region of the UK where they live
- industry of the company that they work for
- company size
- parenthood status

Table 1. The odds ratio of being chosen for promotion, salary increase, and training by working mode: logit models.

Variables	Promotion	Salary increase	Training
Working mode: Hybrid	0.706***	0.725***	0.994
	(0.070)	(0.071)	(0.098)
Working mode: Home	0.617***	0.656***	0.753***
	(0.063)	(0.066)	(0.072)
Sex: Women	1.600***	1.632***	1.147*
	(0.129)	(0.134)	(0.093)
Family situation: 1 child	1.073	1.040	0.996
	(0.106)	(0.103)	(0.096)
Family situation: 3 children	1.071	1.286***	0.986
	(0.105)	(0.124)	(0.092)
Age: 40 years old	1.101	1.010	0.893
	(0.111)	(0.102)	(0.088)
Age: 41 years old	1.146	1.017	1.000
	(0.114)	(0.099)	(0.099)
Work experience: 13 years	1.591***	1.477***	0.729***
	(0.129)	(0.120)	(0.059)
Skills: social 4, analytical 1	0.215***	0.207***	3.703***
	(0.023)	(0.021)	(0.383)
Skills: social 3, analytical 2	0.393***	0.384***	2.020***
	(0.039)	(0.038)	(0.194)
Constant	1.645***	1.709***	0.643***
	(0.207)	(0.220)	(0.078)
Pseudo R-Squared	0.087	0.089	0.057
Observations	2,804	2,804	2,804

*** p<0.01, ** p<0.05, * p<0.1; SE in parentheses

Table 2. The odds ratio of being chosen for promotion, salary increase, and training by the interaction of working mode and prevalence of WFH in the team: logit models.

Variables	Promotion	Salary increase	Training
Working mode: Hybrid	0.706***	0.725***	0.994
	(0.070)	(0.071)	(0.098)
Working mode: Home	0.617***	0.656***	0.753***
	(0.063)	(0.066)	(0.072)
WFH prevalence: Moderate	0.762	0.715**	0.791
	(0.130)	(0.119)	(0.127)
WFH prevalence: High	0.536***	0.651***	0.912
	(0.075)	(0.090)	(0.122)
Working mode: Hybrid # WFH prevalence: Moderate	1.301	1.249	1.528
	(0.375)	(0.351)	(0.438)
Working mode: Hybrid # WFH prevalence: High	2.326***	1.474	1.108
	(0.552)	(0.356)	(0.261)
Working mode: Home # WFH prevalence: Moderate	1.785*	2.217***	1.330
	(0.532)	(0.660)	(0.375)
Working mode: Home # WFH prevalence: High	2.892***	2.477***	1.180
	(0.712)	(0.599)	(0.274)
Sex: Women	1.606***	1.632***	1.148*
	(0.130)	(0.135)	(0.093)
Family situation: 1 child	1.089	1.054	1.000
	(0.108)	(0.104)	(0.097)
Family situation: 3 children	1.091	1.302***	0.982
	(0.108)	(0.127)	(0.092)
Age: 40 years old	1.117	1.026	0.897
	(0.114)	(0.104)	(0.088)
Age: 41 years old	1.147	1.013	0.999
	(0.114)	(0.098)	(0.099)
Work experience: 13 years	1.587***	1.477***	0.730***

	(0.129)	(0.121)	(0.059)
Skills: social 4, analytical 1	0.210***	0.203***	3.697***
	(0.023)	(0.021)	(0.383)
Skills: social 3, analytical 2	0.386***	0.380***	2.030***
	(0.039)	(0.038)	(0.195)
Constant	2.382***	2.274***	0.706**
	(0.382)	(0.371)	(0.108)
Pseudo R-Squared	0.093	0.093	0.057
Observations	2,804	2,804	2,804

Notes: The prevalence of WFH in the team is measured by the question ‘How many of the workers under your supervision work from home at least one day a week on a regular basis?’ with answers coded into three levels <40% (Low WFH), 40-79% (Moderate WFH) and 80+% (High WFH); SE in parentheses
*** p<0.01, ** p<0.05, * p<0.1

Table 3. The odds ratio of being chosen for promotion, salary increase, and training by the interaction of working mode and manager's frequency of WFH: logit models.

Variables	Promotion	Salary increase	Training
Working mode: Hybrid	0.394***	0.451***	1.198
	(0.087)	(0.102)	(0.270)
Working mode: Home	0.334***	0.380***	0.634**
	(0.075)	(0.089)	(0.134)
Manager's frequency of WFH: Sporadically	0.775	0.706*	0.894
	(0.152)	(0.134)	(0.163)
Manager's frequency of WFH: Often	0.562***	0.636***	1.043
	(0.084)	(0.097)	(0.148)
Working mode: Hybrid # Manager's frequency of WFH: Sporadically	1.273	1.663	1.117
	(0.405)	(0.532)	(0.359)
Working mode: Hybrid # Manager's frequency of WFH: Often	2.424***	1.890**	0.717
	(0.615)	(0.489)	(0.185)
Working mode: Home # Manager's frequency of WFH: Sporadically	1.481	1.489	1.372
	(0.506)	(0.498)	(0.441)
Working mode: Home # Manager's frequency of WFH: Often	2.461***	2.186***	1.214
	(0.637)	(0.581)	(0.294)
Sex: Women	1.600***	1.632***	1.148*
	(0.130)	(0.134)	(0.093)
Family situation: 1 child	1.079	1.044	0.995
	(0.107)	(0.103)	(0.096)
Family situation: 3 children	1.081	1.287***	0.977
	(0.107)	(0.125)	(0.092)
Age: 40 years old	1.118	1.022	0.895
	(0.113)	(0.104)	(0.088)
Age: 41 years old	1.159	1.027	0.999
	(0.115)	(0.100)	(0.099)
Work experience: 13 years	1.586***	1.472***	0.729***
	(0.129)	(0.121)	(0.059)

Skills: social 4, analytical 1	0.212***	0.204***	3.688***
	(0.023)	(0.021)	(0.383)
Skills: social 3, analytical 2	0.391***	0.382***	2.011***
	(0.040)	(0.038)	(0.194)
Constant	2.422***	2.391***	0.643***
	(0.425)	(0.434)	(0.105)
Pseudo R-Squared	0.092	0.092	0.058
Observations	2,804	2,804	2,804

Notes: The prevalence of WFH in the team is measured by the question 'How many of the workers under your supervision work from home at least one day a week on a regular basis?' with answers coded into three levels <40% (Low WFH), 40-79% (Moderate WFH) and 80+% (High WFH); SE in parentheses
*** p<0.01, ** p<0.05, * p<0.1

Table 4. The odds ratio of being chosen for promotion, salary increase, and training by the interaction of working mode, gender and parenthood status: logit models.

Variables	Promotion	Salary increase	Training
Working mode: Hybrid	0.534***	0.578**	1.081
	(0.121)	(0.133)	(0.241)
Working mode: Home	0.466***	0.663*	0.812
	(0.113)	(0.159)	(0.187)
Parenthood status: parents	0.903	1.073	0.942
	(0.171)	(0.208)	(0.180)
Working mode: Hybrid # Parents	1.264	1.096	1.255
	(0.359)	(0.321)	(0.362)
Working mode: Home # Parents	1.197	0.898	1.077
	(0.351)	(0.264)	(0.317)
Sex: Women	1.335	1.533*	1.458*
	(0.303)	(0.349)	(0.310)
Working mode: Hybrid # Women	1.208	0.972	0.731
	(0.396)	(0.322)	(0.237)
Working mode: Home # Women	1.251	0.925	0.795
	(0.425)	(0.311)	(0.261)
Working mode: Hybrid # Parents # Women	1.106	1.753	0.795
	(0.442)	(0.720)	(0.322)
Working mode: Home # Parents # Women	1.152	1.369	0.966
	(0.468)	(0.555)	(0.389)
Age: 40 years old	1.102	1.026	0.892
	(0.112)	(0.104)	(0.087)
Age: 41 years old	1.142	1.030	0.999
	(0.114)	(0.100)	(0.099)
Work experience: 13 years	1.593***	1.495***	0.725***
	(0.129)	(0.122)	(0.059)
Skills: social 4, analytical 1	0.215***	0.207***	3.736***
	(0.023)	(0.021)	(0.389)
Skills: social 3, analytical 2	0.393***	0.383***	2.029***
	(0.040)	(0.038)	(0.196)
Constant	2.011***	1.898***	0.593***
	(0.342)	(0.336)	(0.098)
Pseudo R-Squared	0.089	0.090	0.058
Observations	2,804	2,804	2,804

*** p<0.01, ** p<0.05, * p<0.1; SE in parentheses

Fig 1. The predicted probabilities for being chosen for promotion by the interaction of working mode, gender, parenthood status and the prevalence of WFH in the team: logit models.

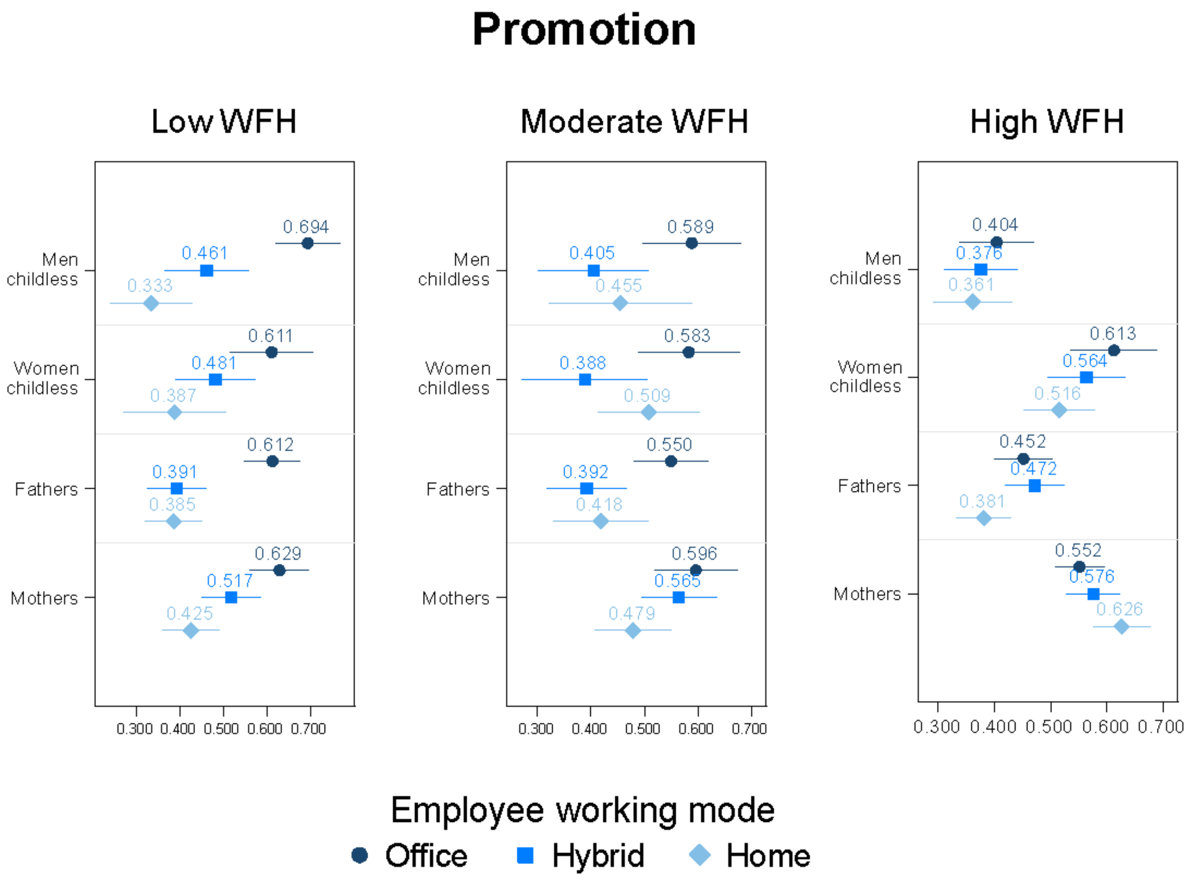

Notes: The prevalence of WFH in the team is measured by the question 'How many of the workers under your supervision work from home at least one day a week on a regular basis?' with answers coded into three levels <40% (Low WFH), 40-79% (Moderate WFH) and 80+% (High WFH).

Fig 2. The predicted probabilities for being chosen for salary raise by the interaction of working mode, gender, parenthood status and the prevalence of WFH in the team: logit models.

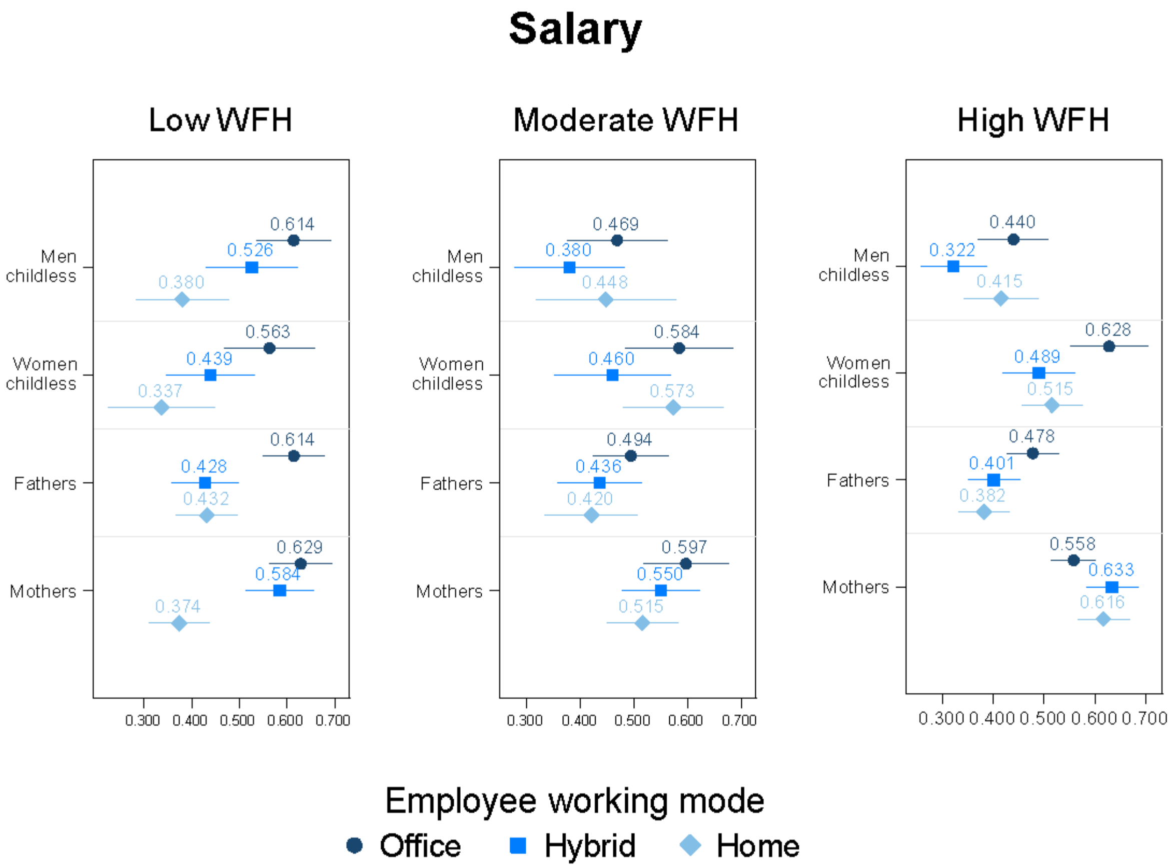

Notes: The same as in Fig 1

Fig 3. The predicted probabilities for being chosen for training by the interaction of working mode, gender, parenthood status and the prevalence of WFH in the team: logit models.

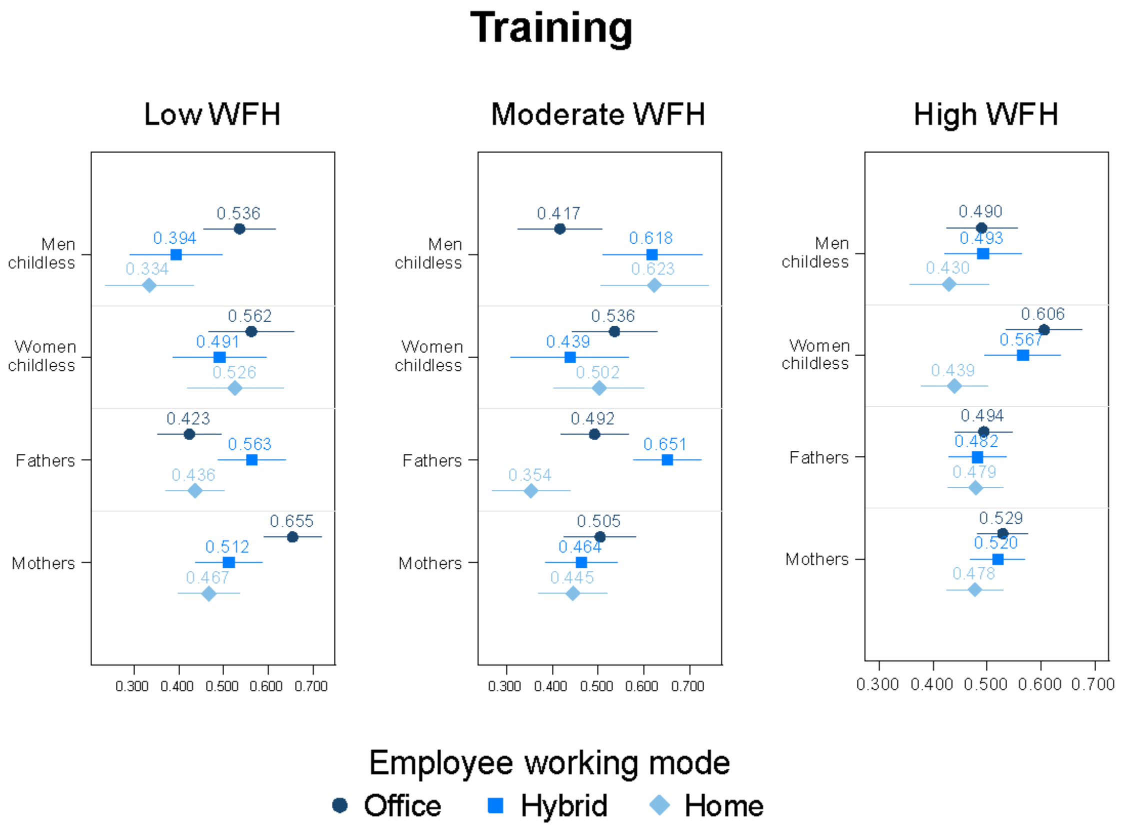

Notes: The same as in Fig 1

Table 5. The odds ratio of being chosen for promotion, salary increase, and training by the interaction of working mode, gender, parenthood status and the prevalence of WFH in the team: logit models.

Variables	Promotion	Salary increase	Training
Working mode: Hybrid	0.340**	0.670	0.539
	(0.149)	(0.297)	(0.226)
Working mode: Home	0.186***	0.345**	0.407**
	(0.088)	(0.155)	(0.181)
Parenthood status: Parents	0.672	1.001	0.614
	(0.248)	(0.362)	(0.221)
Working mode: Hybrid # Parents	1.080	0.643	3.399**
	(0.586)	(0.356)	(1.861)
Working mode: Home # Parents	1.914	1.270	2.602*
	(1.073)	(0.684)	(1.437)
Sex: Women	0.668	0.792	1.122
	(0.286)	(0.318)	(0.440)
Working mode: Hybrid # Women	1.638	0.855	1.365
	(1.017)	(0.523)	(0.848)
Working mode: Home # Women	1.946	1.024	2.099
	(1.414)	(0.708)	(1.357)
Parents # Women	1.619	1.352	2.478*
	(0.852)	(0.675)	(1.246)
Working mode: Hybrid # Parents # Women	0.999	2.209	0.211**
	(0.737)	(1.671)	(0.162)
Working mode: Home # Parents # Women	0.571	0.697	0.196**
	(0.469)	(0.550)	(0.150)
WFH prevalence: Moderate	0.602	0.518	0.596
	(0.249)	(0.211)	(0.232)
WFH prevalence: High	0.262***	0.455**	0.820
	(0.093)	(0.160)	(0.267)
Working mode: Hybrid # WFH prevalence: Moderate	1.282	0.993	4.490**

	(0.821)	(0.639)	(2.889)
Working mode: Hybrid # WFH prevalence: High	2.573*	0.854	1.877
	(1.416)	(0.478)	(0.983)
Working mode: Home # WFH prevalence: Moderate	2.943	2.638	6.076***
	(2.171)	(1.888)	(4.135)
Working mode: Home # WFH prevalence: High	4.384**	2.592*	1.893
	(2.552)	(1.451)	(1.039)
Parents # WFH prevalence: Moderate	1.247	1.121	2.263
	(0.663)	(0.608)	(1.200)
Parents # WFH prevalence: High	1.848	1.186	1.659
	(0.855)	(0.548)	(0.755)
Working mode: Hybrid # Parents # WFH prevalence: Moderate	1.042	1.795	0.247*
	(0.839)	(1.485)	(0.207)
Working mode: Hybrid # Parents # WFH prevalence: High	1.159	1.917	0.276*
	(0.787)	(1.332)	(0.188)
Working mode: Home # Parents # WFH prevalence: Moderate	0.528	0.620	0.084***
	(0.464)	(0.533)	(0.072)
Working mode: Home # Parents # WFH prevalence: High	0.463	0.568	0.467
	(0.322)	(0.386)	(0.322)
Women # WFH prevalence: Moderate	1.459	2.121	1.498
	(0.903)	(1.286)	(0.861)
Women # WFH prevalence: High	3.851**	2.963**	1.483
	(2.137)	(1.587)	(0.752)
Working mode: Hybrid # Women # WFH prevalence: Moderate	0.581	1.005	0.198*
	(0.531)	(0.921)	(0.182)
Working mode: Hybrid # Women # WFH prevalence: High	0.559	1.091	0.608
	(0.444)	(0.858)	(0.471)
Working mode: Home # Women # WFH prevalence: Moderate	0.671	1.023	0.166*
	(0.687)	(1.024)	(0.156)
Working mode: Home # Women # WFH prevalence: High	0.405	0.655	0.298

	(0.352)	(0.550)	(0.238)
Parents # Women # WFH prevalence: Moderate	0.783	0.700	0.254*
	(0.592)	(0.535)	(0.188)
Parents # Women # WFH prevalence: High	0.376	0.452	0.282**
	(0.250)	(0.291)	(0.181)
Working mode: Hybrid # Parents # Women # WFH prevalence: Moderate	1.862	0.554	7.211*
	(2.079)	(0.644)	(8.414)
Working mode: Hybrid # Parents # Women # WFH prevalence: High	1.115	0.975	5.763*
	(1.053)	(0.940)	(5.531)
Working mode: Home # Parents # Women # WFH prevalence: Moderate	1.426	1.323	20.837***
	(1.718)	(1.592)	(24.081)
Working mode: Home # Parents # Women # WFH prevalence: High	4.327	4.341	6.966**
	(4.338)	(4.234)	(6.649)
Age: 40 years old	1.116	1.034	0.921
	(0.114)	(0.106)	(0.091)
Age: 41 years old	1.130	1.010	1.027
	(0.114)	(0.100)	(0.103)
Work experience: 13 years	1.588***	1.500***	0.721***
	(0.130)	(0.124)	(0.059)
Skills: social 4, analytical 1	0.208***	0.202***	3.779***
	(0.023)	(0.021)	(0.398)
Skills: social 3, analytical 2	0.377***	0.369***	2.050***
	(0.039)	(0.037)	(0.202)
Constant	4.262***	3.215***	0.710
	(1.242)	(0.919)	(0.192)
Pseudo R-Squared	0.100	0.101	0.066
Observations	2,804	2,804	2,804

Notes: The same as in Table 2
*** p<0.01, ** p<0.05, * p<0.1

Table 6. The odds ratio of being chosen for promotion, salary increase, and training by the interaction of working mode, gender, parenthood status and manager's frequency of WFH: logit models.

Variables	Promotion	Salary increase	Training
Working mode: Hybrid	0.330**	0.497	1.556
	(0.186)	(0.291)	(0.843)
Working mode: Home	0.269**	0.727	0.363*
	(0.152)	(0.407)	(0.196)
Parenthood status: Parents	1.029	1.533	0.672
	(0.456)	(0.697)	(0.294)
Working mode: Hybrid # Parents	0.889	0.546	1.214
	(0.589)	(0.373)	(0.808)
Working mode: Home # Parents	1.377	0.630	2.933
	(0.925)	(0.413)	(1.959)
Sex: Women	0.885	1.148	1.266
	(0.451)	(0.565)	(0.572)
Working mode: Hybrid # Women	1.621	1.106	0.546
	(1.215)	(0.845)	(0.408)
Working mode: Home # Women	1.392	0.523	1.813
	(1.059)	(0.382)	(1.287)
Parents # Women	1.328	1.018	1.726
	(0.804)	(0.609)	(1.007)
Working mode: Hybrid # Parents # Women	1.062	2.227	0.797
	(0.926)	(2.022)	(0.731)
Working mode: Home # Parents # Women	0.656	1.121	0.241*
	(0.590)	(0.998)	(0.208)
Manager's frequency of WFH: Sporadically	1.169	1.157	0.859
	(0.597)	(0.582)	(0.393)
Manager's frequency of WFH: Often	0.489*	0.697	0.888
	(0.196)	(0.288)	(0.324)
Working mode: Hybrid # Manager's frequency of WFH: Sporadically	0.762	1.136	0.894
	(0.620)	(0.891)	(0.699)

Working mode: Hybrid # Manager's frequency of WFH: Often	2.288	1.252	0.600
	(1.434)	(0.818)	(0.364)
Working mode: Home # Manager's frequency of WFH: Sporadically	1.010	0.444	2.114
	(0.854)	(0.354)	(1.668)
Working mode: Home # Manager's frequency of WFH: Often	2.396	1.062	2.908*
	(1.532)	(0.675)	(1.773)
Parents # Manager's frequency of WFH: Sporadically	0.650	0.498	1.424
	(0.420)	(0.320)	(0.887)
Parents # Manager's frequency of WFH: Often	0.889	0.667	1.570
	(0.448)	(0.345)	(0.785)
Working mode: Hybrid # Parents # Manager's frequency of WFH: Sporadically	1.538	2.599	0.923
	(1.509)	(2.498)	(0.903)
Working mode: Hybrid # Parents # Manager's frequency of WFH: Often	1.643	2.396	1.015
	(1.233)	(1.866)	(0.768)
Working mode: Home # Parents # Manager's frequency of WFH: Sporadically	1.516	3.991	0.311
	(1.509)	(3.814)	(0.302)
Working mode: Home # Parents # Manager's frequency of WFH: Often	0.723	1.231	0.277*
	(0.554)	(0.930)	(0.211)
Women # Manager's frequency of WFH: Sporadically	1.196	1.019	0.789
	(0.864)	(0.710)	(0.507)
Women # Manager's frequency of WFH: Often	1.774	1.596	1.414
	(1.052)	(0.928)	(0.756)
Working mode: Hybrid # Women # Manager's frequency of WFH: Sporadically	0.787	0.559	1.972
	(0.863)	(0.601)	(2.200)
Working mode: Hybrid # Women # Manager's frequency of WFH: Often	0.694	0.911	1.214
	(0.598)	(0.801)	(1.036)
Working mode: Home # Women # Manager's frequency of WFH: Sporadically	0.526	1.564	1.214
	(0.608)	(1.787)	(1.302)

Working mode: Home # Women # Manager's frequency of WFH: Often	1.003	2.142	0.249*
	(0.877)	(1.822)	(0.206)
Parents # Women # Manager's frequency of WFH: Sporadically	0.475	0.793	0.811
	(0.411)	(0.664)	(0.666)
Parents # Women # Manager's frequency of WFH: Often	0.809	0.844	0.403
	(0.570)	(0.591)	(0.277)
Working mode: Hybrid # Parents # Women # Manager's frequency of WFH: Sporadically	2.854	1.205	0.787
	(3.660)	(1.539)	(1.063)
Working mode: Hybrid # Parents # Women # Manager's frequency of WFH: Often	0.763	0.670	1.177
	(0.777)	(0.709)	(1.242)
Working mode: Home # Parents # Women # Manager's frequency of WFH: Sporadically	3.827	1.188	2.074
	(5.062)	(1.533)	(2.644)
Working mode: Home # Parents # Women # Manager's frequency of WFH: Often	1.786	1.439	7.987**
	(1.859)	(1.492)	(8.072)
Age: 40 years old	1.109	1.030	0.897
	(0.114)	(0.105)	(0.089)
Age: 41 years old	1.136	1.012	0.998
	(0.115)	(0.100)	(0.100)
Work experience: 13 years	1.596***	1.500***	0.724***
	(0.131)	(0.124)	(0.059)
Skills: social 4, analytical 1	0.211***	0.203***	3.725***
	(0.023)	(0.021)	(0.391)
Skills: social 3, analytical 2	0.385***	0.372***	2.039***
	(0.040)	(0.037)	(0.200)
Constant	3.102***	2.360**	0.655
	(1.153)	(0.907)	(0.219)
Pseudo R-Squared	0.099	0.101	0.063
Observations	2,804	2,804	2,804

Notes: The same as in Table 2 *** p<0.01, ** p<0.05, * p<0.1

Fig 4. The predicted probabilities for being chosen for promotion by the interaction of working mode, gender, parenthood status and the manager's frequency of WFH: logit models.

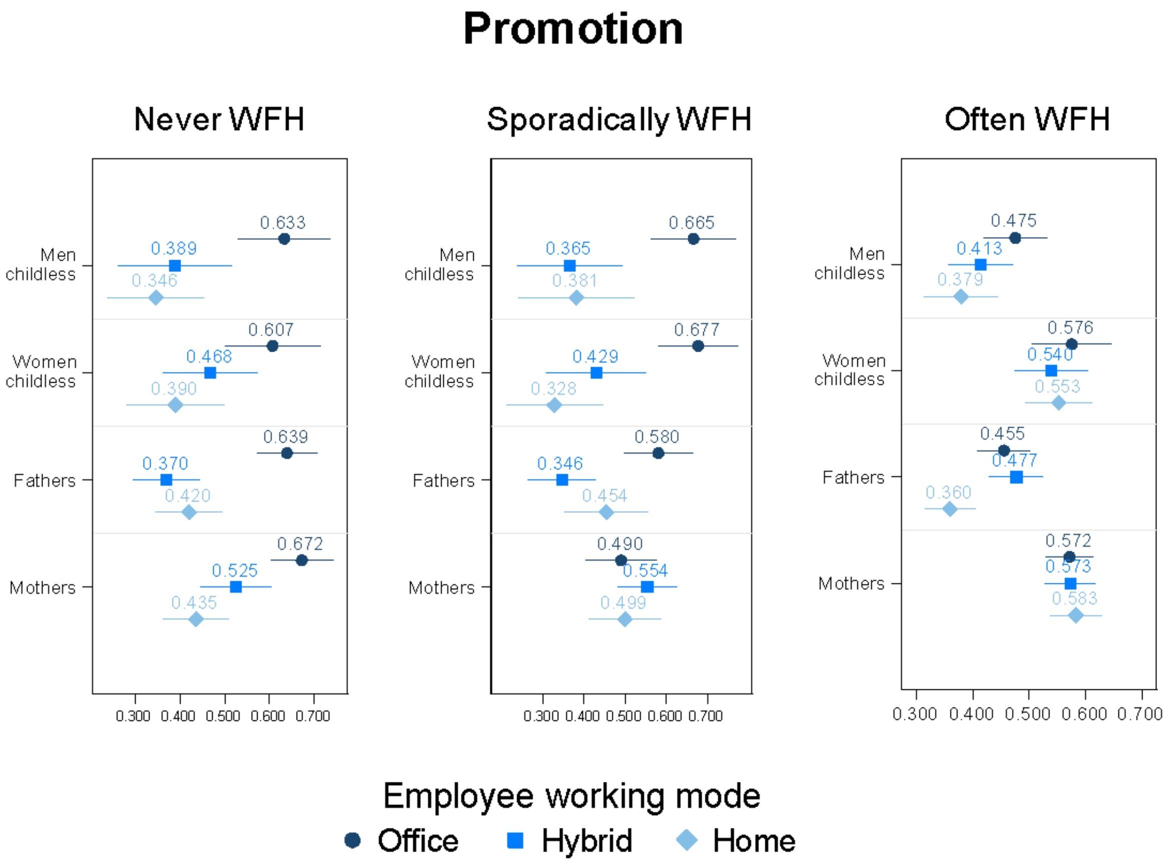

Notes: The manager's frequency of WFH is measured by the questions *'Do you currently work from home at least from time to time?'* and *'How often do you currently work from home?'*, with answers coded into three levels: Never, Sporadically (several times a month or less often), and Often (several times a week or daily).

Fig 5. The predicted probabilities for being chosen for salary raise by the interaction of working mode, gender, parenthood status and the manager's frequency of WFH: logit models.

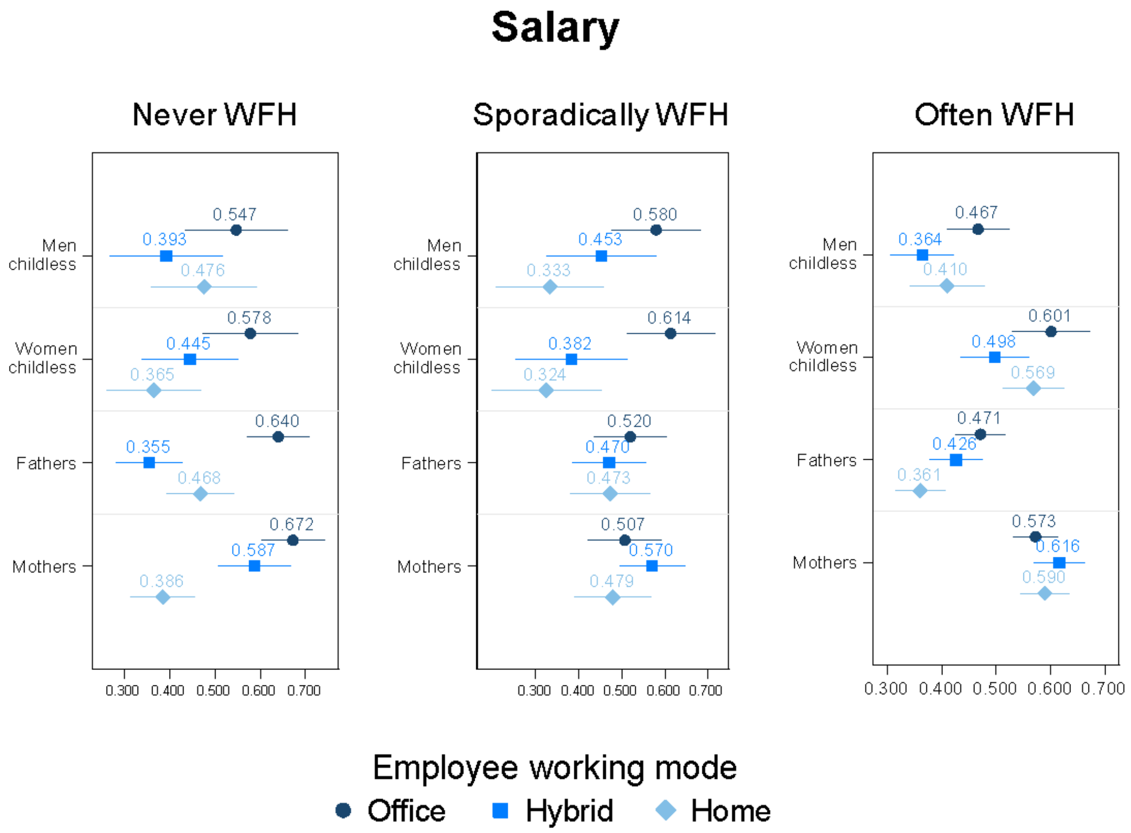

Notes: The same as in Fig 4

Fig 6. The predicted probabilities for being chosen for training by the interaction of working mode, gender, parenthood status and the manager's frequency of WFH: logit models.

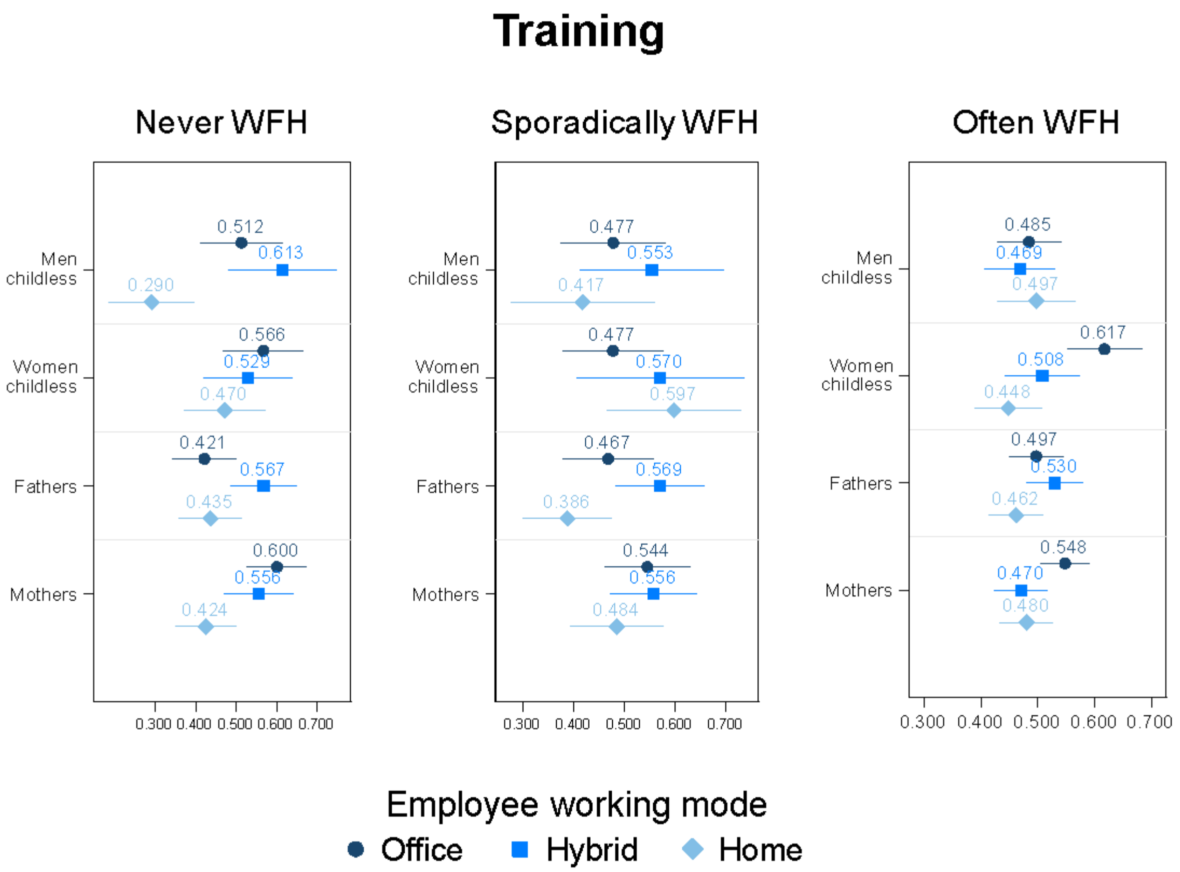

Notes: The same as in Fig 4
